# *GIGYF1* disruption associates with autism and impaired IGF-1R signaling

**DOI:** 10.1172/JCI159806

**Published:** 2022-10-03

**Authors:** Guodong Chen, Bin Yu, Senwei Tan, Jieqiong Tan, Xiangbin Jia, Qiumeng Zhang, Xiaolei Zhang, Qian Jiang, Yue Hua, Yaoling Han, Shengjie Luo, Kendra Hoekzema, Raphael A. Bernier, Rachel K. Earl, Evangeline C. Kurtz-Nelson, Michaela J. Idleburg, Suneeta Madan-Khetarpal, Rebecca Clark, Jessica Sebastian, Alberto Fernandez-Jaen, Sara Alvarez, Staci D. King, Luiza L.P. Ramos, Mara Lucia S.F. Santos, Donna M. Martin, Dan Brooks, Joseph D. Symonds, Ioana Cutcutache, Qian Pan, Zhengmao Hu, Ling Yuan, Evan E. Eichler, Kun Xia, Hui Guo

**Affiliations:** 1 Center for Medical Genetics and Hunan Key Laboratory of Medical Genetics, School of Life Sciences, Central South University, Changsha, Hunan, China.; 2 Department of Genome Sciences, University of Washington School of Medicine, Seattle, Washington, USA.; 3 Department of Psychiatry, University of Washington, Seattle, Washington, USA.; 4 Department of Medical Genetics, University of Pittsburgh Medical Center (UPMC) Children’s Hospital of Pittsburgh, Pittsburgh, Pennsylvania, USA.; 5 Department of Human Genetics, University of Pittsburgh, Pittsburgh, Pennsylvania, USA.; 6 Department of Pediatric Neurology, Hospital Universitario Quirónsalud, School of Medicine, Universidad Europea de Madrid, Madrid, Spain.; 7 Department of Genomics and Medicine, Genomics and Medicine, NIMGenetics, Madrid, Spain.; 8 Department of Neurology, Baylor College of Medicine, Houston, Texas, USA.; 9 Mendelics Genomic Analysis, Sao Paulo, Brazil.; 10 Neurogenetics Unit, Department of Neurology, University of Sao Paulo, Sao Paulo, Brazil.; 11 Neuropediatric Division, Hospital Pequeno Principe, Curitiba, Paraná, Brazil.; 12 Department of Pediatrics, Michigan Medicine, University of Michigan, Ann Arbor, Michigan, USA.; 13 Paediatric Neurosciences Research Group, Royal Hospital for Children, Glasgow, United Kingdom.; 14 Translational Medicine, UCB Pharma, Slough, United Kingdom.; 15 Howard Hughes Medical Institute, University of Washington, Seattle, Washington, USA.; 16 CAS Center for Excellence in Brain Science and Intelligences Technology (CEBSIT), Chinese Academy of Sciences, Shanghai, China.; 17 Hengyang Medical School, University of South China, Hunan, China.; 18 Hunan Key Laboratory of Animal Models for Human Diseases, Changsha, Hunan, China.

**Keywords:** Genetics, Neuroscience, Molecular genetics, Neurodevelopment, Neurological disorders

## Abstract

Autism spectrum disorder (ASD) represents a group of neurodevelopmental phenotypes with a strong genetic component. An excess of likely gene-disruptive (LGD) mutations in *GIGYF1* was implicated in ASD. Here, we report that *GIGYF1* is the second-most mutated gene among known ASD high–confidence risk genes. We investigated the inheritance of 46 *GIGYF1* LGD variants, including the highly recurrent mutation c.333del:p.L111Rfs*234. Inherited *GIGYF1* heterozygous LGD variants were 1.8 times more common than de novo mutations. Among individuals with ASD, cognitive impairments were less likely in those with *GIGYF1* LGD variants relative to those with other high-confidence gene mutations. Using a *Gigyf1* conditional KO mouse model, we showed that haploinsufficiency in the developing brain led to social impairments without significant cognitive impairments. In contrast, homozygous mice showed more severe social disability as well as cognitive impairments. *Gigyf1* deficiency in mice led to a reduction in the number of upper-layer cortical neurons, accompanied by a decrease in proliferation and increase in differentiation of neural progenitor cells. We showed that GIGYF1 regulated the recycling of IGF-1R to the cell surface. KO of GIGYF1 led to a decreased level of IGF-1R on the cell surface, disrupting the IGF-1R/ERK signaling pathway. In summary, our findings show that GIGYF1 is a regulator of IGF-1R recycling. Haploinsufficiency of *GIGYF1* was associated with autistic behavior, likely through interference with IGF-1R/ERK signaling pathway.

## Graphical abstract

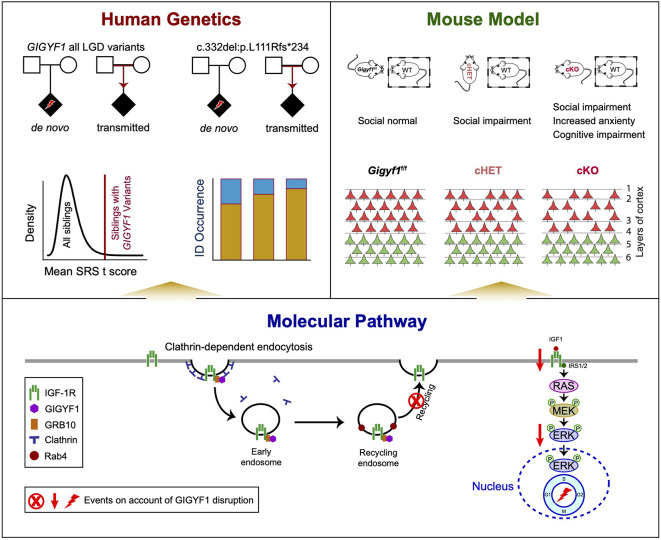

## Introduction

Autism spectrum disorder (ASD) is a highly heritable neurodevelopmental condition characterized by impaired social communication and repetitive behaviors (1). Previous studies have identified over 200 high-confidence ASD risk genes — most of which are based on significant enrichments of de novo variants in families with ASD (https://gene.sfari.org/). Besides ASD, the majority of high-confidence genes are also enriched for de novo variants in other neurodevelopment disorders (NDDs) associated with intellectual disability and developmental delay (DD) (2, 3). Because of the extreme rarity of the pathogenic variants within these high-penetrance genes, the exact mutation and inheritance patterns and detailed phenotypic associations of most ASD-risk genes are still largely unknown.

*GIGYF1* encodes a protein involved in the insulin-like growth factor receptor (IGF-R) signaling pathway (4). The first evidence for its role in ASD risk was the identification of 3 de novo likely gene-disruptive variants (LGD variants) in 3 unrelated individuals with ASD by screening over 2,500 families from the Simons Simplex Collection (SSC) cohort (5, 6). A recent study using a larger sample size confirms a genome-wide significant enrichment of *GIGYF1* de novo LGD variants in individuals with ASD (7). In addition, an excess of de novo LGD variants in *GIGYF1* was also recently reported in DD (3). Despite these findings, its inheritance among families with ASD and phenotypic association of this specific gene are still not well characterized. More importantly, how *GIGYF1* is involved in the mechanisms underpinning neurodevelopment and autistic behavior is unknown.

In this study, we combine human genetics, conditional KO models and molecular studies to highlight the role of *GIGYF1* in the neurobiology of ASD. We report an excess of de novo LGD mutations and transmission disequilibrium of inherited *GIGYF1* LGD mutations among individuals with ASD and low cognitive impairment, and we discovered ASD endophenotypes among carriers without an ASD diagnosis. We show that haploinsufficiency of *Gigyf1* in mice leads to social and behavioral disabilities without substantial cognitive impairments. Mechanistically, disruption of GIGYF1, what we believe to be a new regulator of IGF-1R recycling, leads to inactivation of the IGF-1R/ERK signaling pathway, and this likely affects neurodevelopment and autistic behaviors by disrupting the neural progenitor cell (NPC) cycle dynamics early in brain development.

## Results

### Mutation pattern and inheritance mode of GIGYF1 heterogeneous LGD variants.

**Figure 1:**
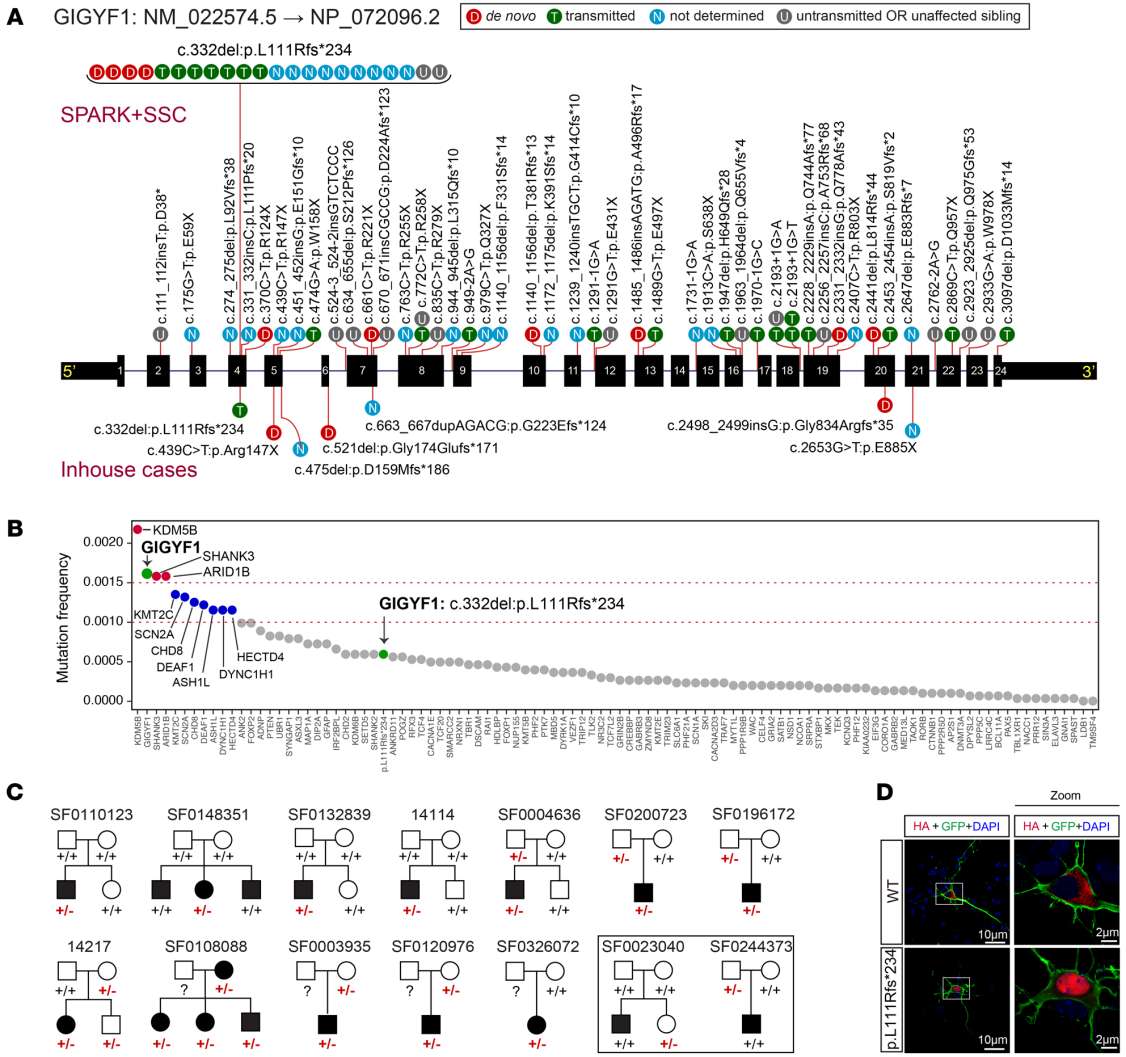
Pattern, distribution, and inheritance of *GIGYF1* heterozygous LGD mutations in humans. (**A**) Mutation pattern of *GIGYF1* likely gene-disruptive variants (LGD variants) identified in SPARK and/or SSC cohorts (above) and through GeneMatcher (below) on a gene model. (**B**) Ranked mutation frequency of LGD variants in 102 high-confidence genes identified in Satterstrom et al. (7). (**C**) Pedigrees with the recurrent variant p.L111Rfs*234 identified in the SPARK and SSC cohorts. +/+, WT; +/–, heterozygous. Families with untransmitted or de novo GIGFY1 LGD variants only in unaffected family members are indicated by the square outline. Solid circles or squares represent individuals with an ASD diagnosis. Numbers above each pedigree are SPARK family designations; red +/- indicate individuals with mutations. (**D**) The recurrent LGD locus p.L111Rfs*234 shows abnormal localization in mouse primary-cultured neurons. The WT plasmid is mainly located in the cytoplasm; however, the mutant plasmid is exclusively located in the nuclei. Scale bars: 10 μm, 2 μm for zoom image.

To comprehensively delineate the mutation pattern and inheritance modes of *GIGYF1* variants in ASD, we analyzed whole-exome sequencing (WES) or whole-genome sequencing (WGS) data from 2 autism cohorts: Simons Foundation Powering Autism Research (SPARK) (8) and the Simons Simplex Collection (SSC) (9). Combined, the collection includes 20,452 families with 1 or both biological parents available (23,351 ASD cases) and 12,227 ASD cases without parental data (Supplemental Table 1; supplemental material available online with this article; https://doi.org/10.1172/JCI159806DS1). We identified 35 high-confidence *GIGYF1* LGD variants in 60 individuals with ASD from 55 families or singleton cases (Figure 1A, Supplemental Figure 1, and Supplemental Table 2). Six of them were de novo, 15 were transmitted, and 1 was de novo or transmitted in different families. The inheritance of the other 13 variants was unknown due to lack of parental data. In addition, we also identified 8 nontransmitted *GIGYF1* LGD variants in parents without ASD diagnoses and 3 LGD variants that were either de novo or transmitted to siblings (without ASD diagnoses) only (Figure 1A, Supplemental Figure 1, and Supplemental Table 2). We found that transmission of LGD mutations was 1.8 times more likely than de novo mutations among families with ASD where both biological parents were available for study. We found a significant de novo enrichment (*P <* 2.7 × 10^–12^) and significant transmission disequilibrium (*P <* 1 × 10^–5^) of *GIGYF1* heterozygous LGD variants in the 2 cohorts. Of note, we found that the frequency of *GIGYF1* LGD variants ranked second among known ASD high-confidence genes (Figure 1B).

Interestingly, a recurrent LGD variant (c.332del:p.L111Rfs*234) was detected in 23 individuals with ASD from 20 families or singleton cases (Figure 1, A and B). This variant occurred de novo in 4 individuals with ASD and was transmitted in 9 individuals with ASD (Figure 1C). In addition, this variant was also observed in 2 unrelated siblings without any ASD diagnoses (1 inherited and 1 de novo) and 1 unaffected parent who did not transmit this variant to children (Figure 1C). De novo occurrence of this variant in ASD was significantly higher than random occurrence in the general population, even with a gene-level mutation rate used as background (*P* = 0.0004). Significant transmission disequilibrium was also observed for this specific variant (*P* = 0.03). To characterize the functional effect of this variant, we constructed the WT and mutant plasmids and performed immunoblotting and immunostaining in HEK293T cells and HeLa cells. We revealed that the p.L111Rfs*234 variant produced a truncated protein with remarkably increased expression and abnormal localization (Supplemental Figures 2 and 3). The WT protein localizes predominantly to the cytoplasm, in contrast to the mutant, which is restricted to the nucleus. The abnormal localization was also observed in Neuro2a cells (Supplemental Figure 3) and mouse primary cultured neurons (Figure 1D).

In addition to the variants detected in the SPARK and SSC cohorts, we recruited an additional 7 new cases with *GIGYF1* heterozygous LGD variants (3 de novo, 1 inherited, and 3 of unknown inheritance) (Figure 1A) and 1 case with a de novo missense variant through a network of international collaborators connected by GeneMatcher (10), as well as collecting the detailed phenotype information (Supplemental Tables 3 and 4). Five of the 6 individuals with ASD assessments were diagnosed as having ASD. Of note, the recurrent variant p.L111Rfs*234 was transmitted in a family with substantial autism history (Supplemental Figure 4).

### Phenotypic association of GIGYF1 heterozygous LGD variants.

**Figure 2:**
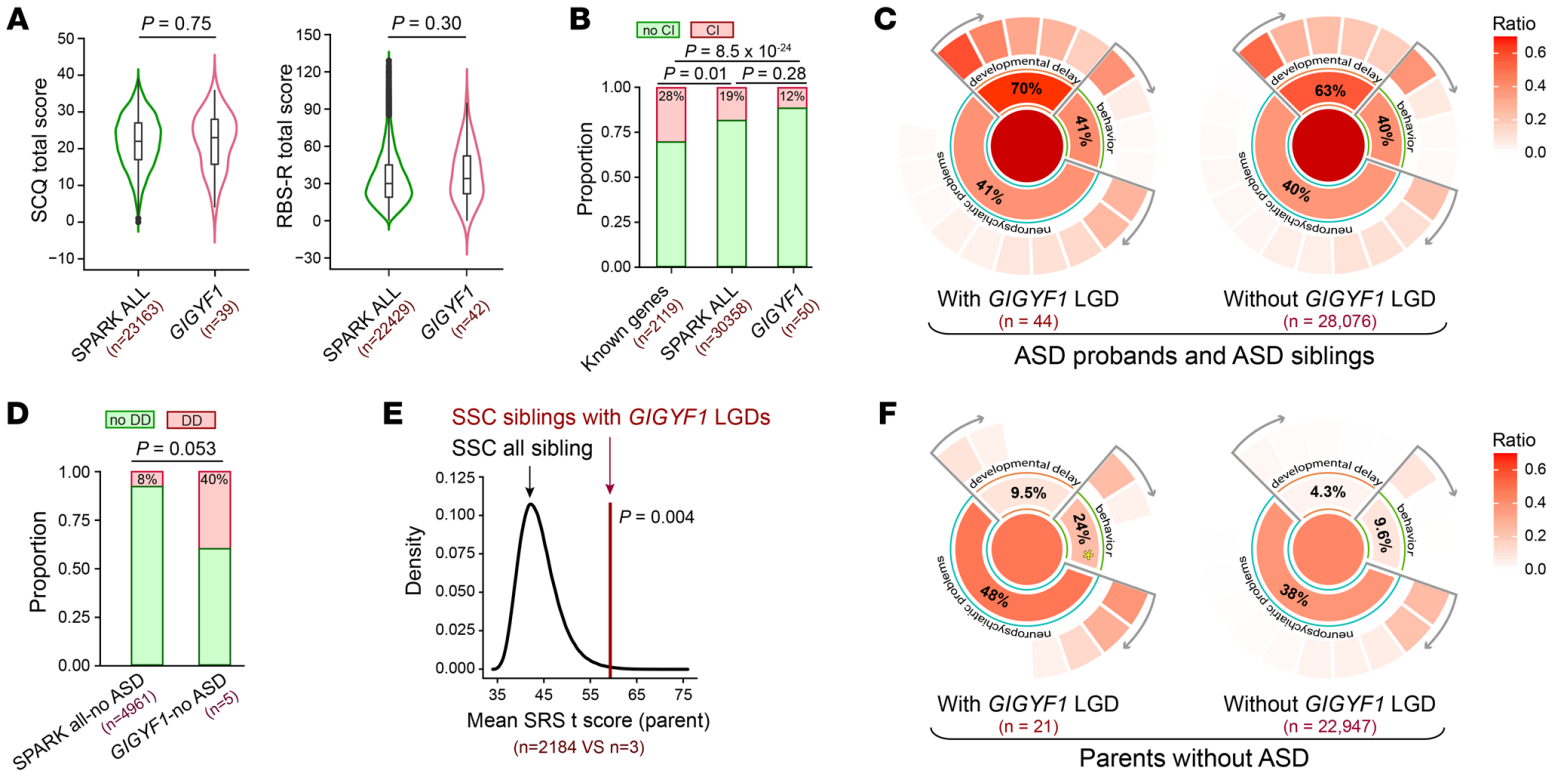
Phenotypic correlation of *GIGYF1* heterozygous LGD mutations. (**A**) Comparison of the SCQ and RBS-R scores in children with ASD with *GIGYF1* LGD variants and all children with ASD in SPARK. (**B**) Comparison of cognitive impairment (CI) occurrence rate among children with ASD with *GIGYF1* LGD variants, children with ASD with LGD variants in known high-confidence genes, and all SPARK children with ASD. (**C**) Comparison of the frequency of behavior problems, developmental delays, and neuropsychiatric problems between children with ASD with and without *GIGYF1* LGD variants. The details of specific phenotype items for each phenotype group in the plot are described in Supplemental Table 9. (**D**) Comparison of developmental delay occurrence rate among children without ASD with *GIGYF1* LGD variants and all children without ASD in SPARK. (**E**) Down sampling analysis of SRS *t* score in siblings without ASD from the SSC cohort. (**F**) Comparison of the frequency of behavior problems, developmental delay, and neuropsychiatric problems between nonASD parents with and without *GIGYF1* LGD variants. The details of specific phenotype items for each phenotype group in the plot are described in Supplemental Table 9.

To delineate *GIGYF1*-associated ASD and NDD phenotypes, we first analyzed the severity of the core symptoms of individuals with ASD with *GIGYF1* LGD variants. We computed the scores on 2 scales, the Social Communication Questionnaire (SCQ) and the Repetitive Behaviors Scale–Revised (RBS-R) (Supplemental Table 5), which represent the severity of social communication and repetitive behaviors, respectively. We found that the mean of SCQ (*P* = 0.75) and RBS-R (*P* = 0.30) scores of individuals with ASD with *GIGYF1* LGD variants were comparable to those of all individuals with ASD in the SPARK cohort, indicating that *GIGYF1*-monoallelic LGD variants lead to the average severity level of social impairments and repetitive behaviors (Figure 2A).

Known ASD high-confidence genes associate with substantial cognitive impairments (11). To explore the cognition association with *GIGYF1* heterozygous LGD variants, we compared the occurrence of the parent-reported cognitive impairment in SPARK. Individuals with ASD with *GIGYF1* LGD variants were less likely to have cognitive impairments than those with known high-confidence ASD genes (SFARI gene score = 1) (28% versus 12%, *P* = 8.5 × 10^-24^). Although the difference was not significant, the frequency of cognitive impairment in individuals with ASD with *GIGYF1* LGD variants was lower (12% versus 19%, *P* = 0.28) than in all individuals with ASD in SPARK (Figure 2B and Supplemental Table 5). Individuals with ASD with the recurrent site had a frequency of cognitive impairments similar to that of all individuals with ASD with *GIGYF1* LGD variants (15% versus 12%). These data indicate that *GIGYF1* heterozygous LGD variants may associate with better cognitive outcome. To explore the pattern of co-occurring conditions of *GIGYF1* LGD variants, we compared the rate of the parent-reported behavior problems, DD, and neuropsychiatric problems in the SPARK clinical data set (Supplemental Table 6). We found that the rates of parent-reported behavior problems (40.9% versus 39.8%, *P* = 0.88), DD (70.5% versus 63.4%, *P* = 0.43), and neuropsychiatry problems (38.6% versus 37.8%, *P* = 1) in individuals with ASD with *GIGYF1* LGD variants were all comparable with those in individuals with ASD without *GIGYF1* LGD variants in the SPARK cohort (Figure 2C and Supplemental Table 7).

There were 8 siblings without ASD diagnoses (5 in SPARK and 3 in SSC) who carried *GIGYF1* LGD variants. We found that 2 of the 5 siblings in SPARK had parent-reported language and/or motor developmental delay, which showed a marginally significantly higher rate (40% versus 8%, *P* = 0.053) relative to parent reports of language and motor developmental delay in siblings without ASD and *GIGYF1* LGD variants (Figure 2D), although the sample size was too small to draw a conclusion. For the 3 SSC siblings, we revealed that unaffected siblings in the SSC with *GIGYF1* LGD variants had poorer social ability, as reflected by the Social Responsiveness Scale (SRS) score, than siblings without ASD and *GIGYF1* LGD variants (*P* = 0.004, Figure 2E and Supplemental Table 8). In addition to siblings with *GIGYF1* LGD variants but without ASD diagnoses, we also found 23 parents without ASD diagnosis but with transmitted or nontransmitted *GIGYF1* LGD variants. We compared the co-occurring conditions in these parents (21 of 23 with clinical data available) with those in parents without ASD diagnosis and without *GIGYF1* LGD variants (Figure 2F). We found that the frequency of behavior problems (23.8% versus 9.6%), DD (9.5% versus 4.3%), and neuropsychiatry problems (47.6% versus 38%) were higher (1.3–2.5 times) in parents with *GIGYF1* LGD variants; however, only the behavior problems showed statistical significance (*P* = 0.045, Supplemental Table 7) under the current small sample size. These data indicate that *GIGYF1* heterozygous LGD variants might also associate with ASD/NDD endophenotypes in children without ASD diagnosis. Larger sample sizes are needed to further confirm this association.

### Gigyf1 haploinsufficiency in the nervous system leads to social and behavior impairments in mice.

To explore the functions of *GIGYF1* in the developing brain and autistic behavior in vivo, we knocked out exons 1–9 of *Gigyf1* and generated floxed (*Gigyf1^fl/fl^*) mice with conditional alleles carrying loxP sites in introns 1 and 9 by the CRISPR/Cas9 targeting strategy (Supplemental Figure 5A). The *Gigyf1^fl/fl^* mice were crossed with Nestin-Cre mice to generate *Gigyf1^fl/w^*-Cre*^Nestin^* (cHET) and *Gigyf1^fl/fl^*-Cre*^Nestin^* (cKO) mice (Supplemental Figure 5B). Both *Gigyf1* cHET and cKO mice were viable. Immunoblot analysis showed that the cHET mice were haploinsufficient for Gigyf1, and the cKO mice lost Gigyf1 expression (Supplemental Figure 5C). We found that the body weight of the cKO mice was significantly decreased on P30. No difference was observed in the cHET mice (Supplemental Figure 6).

**Figure 3:**
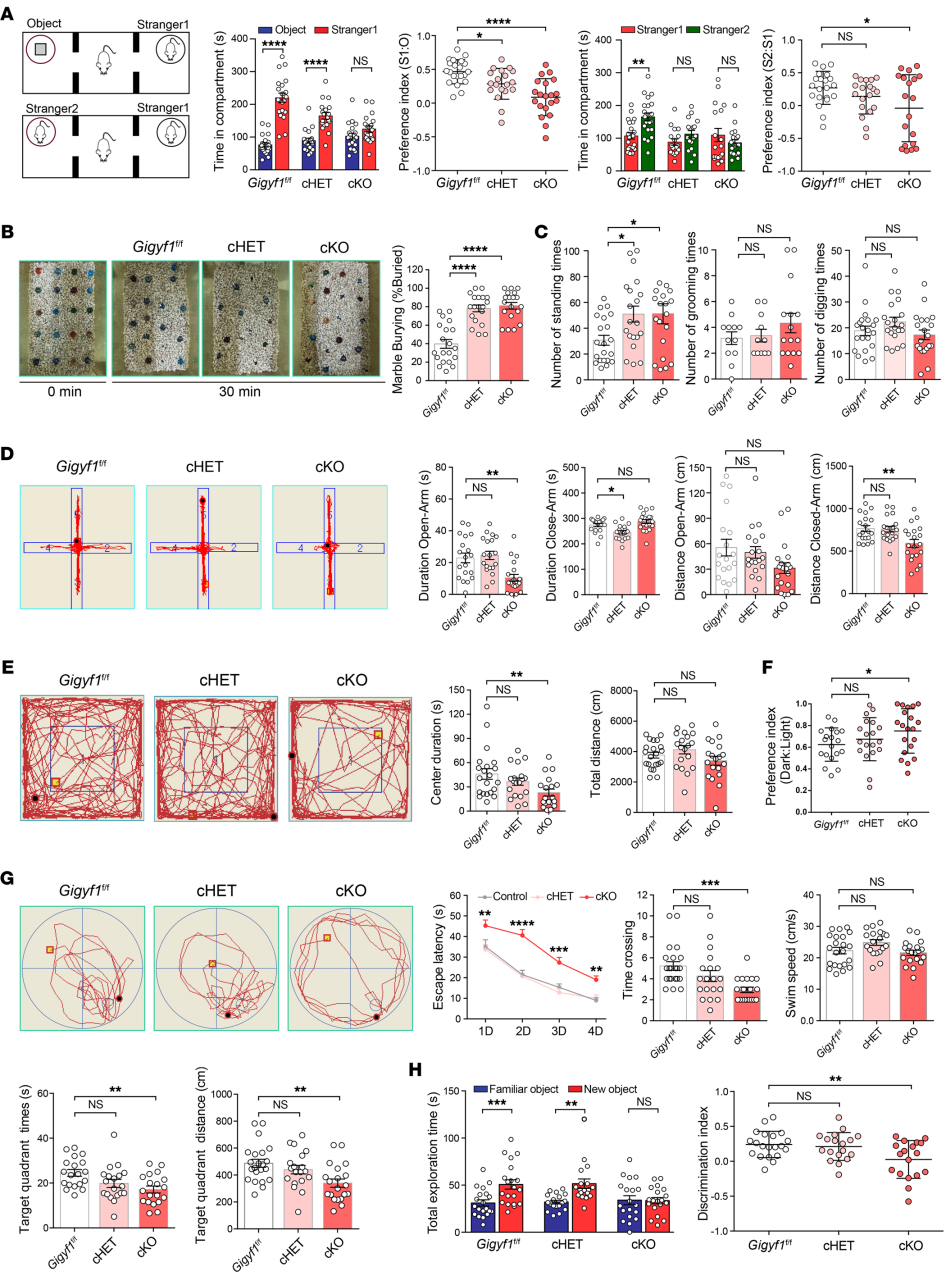
KO and haploinsufficiency of *Gigyf1* in the developing mouse brain results in autism-like behaviors. (**A**) Three-chamber test. The time spent with object (O), stranger 1 (S1) and stranger 2 (S2) was compared. The preference indexes were compared. *n =* 21 (*Gigyf1^fl/fl^*), 19 (cHET), 20 (cKO). Statistical data were analyzed using 1-way ANOVA and 2-tailed Student’s *t* test. (**B**) Marble burying test. The percentage of marbles buried by each mouse was compared. Statistical data were analyzed using 1-way ANOVA. (**C**) Digging, rearing, and grooming test. The numbers of digging, rearing, and grooming incidences of the different groups of mice were compared. *n =* 20 (*Gigyf1^fl/fl^*), 19 (cHET), 19 (cKO). Statistical data were analyzed using 1-way ANOVA. (**D**) Elevated plus-maze test. The time and the total distance in open and closed arms were compared. *n =* 19 (*Gigyf1^fl/fl^*), 19 (cHET), 20 (cKO). Statistical data were analyzed using 1-way ANOVA. (**E**) Open field test. The total distance and center duration were compared. *n =* 21 (*Gigyf1^fl/fl^*), 19 (cHET), 19 (cKO). Statistical data were analyzed using 1-way ANOVA. (**F**) Light-dark box test. The preference indexes to dark box were compared. *n =* 19 (*Gigyf1^fl/fl^*), 19 (cHET), 20 (cKO). Statistical data were analyzed using 1-way ANOVA. (**G**) Morris water maze test. The escape latency in the learning phase, the number of exact crossings over the previously hidden platform in the probe phase, the swim speed, and the time and distance in the target quadrant in the probe phase were compared. *n =* 22 (*Gigyf1^fl/fl^*), 19 (cHET), 20 (cKO). Statistical data were analyzed using 1-way and 2-way ANOVA. (**H**) Novel-object recognition test. Total exploration time and discrimination index were compared. *n =* 20 (*Gigyf1^fl/fl^*), 18 (cHET), 19 (cKO). Statistical data were analyzed using 1-way ANOVA and 2-tailed Student’s *t* test. All data are represented as mean ± SEM. *P < 0.05, **P < 0.01, ***P < 0.001, ****P < 0.0001. cHET, Gigyf1^f/w^-CreNestin; cKO,Gigyf1*^fl/fl^*-CreNestin.

We first performed a 3-chamber social test for voluntary initiation of social interaction and discrimination of social novelty (12). In social interaction tests, *Gigyf1^fl/fl^* — used as a control in the mouse analysis — and cHET mice spent more time with social targets (Figure 3A, Stranger1) than with an inanimate object (Figure 3A). However, the social interaction preference index of cHET mice was significantly lower than that of *Gigyf1^fl/fl^* mice. There was no difference in time spent between social targets and inanimate objects by cKO mice (Figure 3A). In social novelty tests, as expected, *Gigyf1^fl/fl^* mice spent more time with new social targets (Stranger2) than the similar mouse (Stranger1) (Figure 3A). Yet there was no difference in time spent with Stranger2 and Stranger1 for either cHET or cKO mice (Figure 3A). These data suggest that both haploinsufficiency and homozygous KO of *Gigyf1* in the developing brain impair social communication. We then performed a range of tests to evaluate potential repetitive and stereotyped behaviors (Figure 3, B and C). In marble burying tests, both cHET and cKO mice buried more marbles than *Gigyf1^fl/fl^* mice(Figure 3B). We also observed that cHET and cKO mice preferred rearing (Figure 3C). We found no difference in the grooming and digging tests (Figure 3C). Considering that the marble burying test is more likely to reflect anxiety behavior than repetitive behavior and there was no significant difference in the grooming test, these data suggest that haploinsufficiency and homozygous KO of *Gigyf1* in the developing brain has a mild effect on repetitive behaviors in mice.

To determine whether *Gigyf1* deficiency leads to anxiety-like behaviors in mice, we introduced the elevated plus maze test (EPM test), open field test (OF test), and light-dark box test (LDB test). In EPM tests (Figure 3D), the cHET mice displayed similar time and distance in the open arm than *Gigyf1^fl/fl^* mice; however, the cKO mice showed significantly reduced time and travel distance in the open arm than *Gigyf1^fl/fl^* mice. In OF tests (Figure 3E), we also found that the cKO mice tended to stay in the corner and rarely passed through the middle area compared with the *Gigyf1^fl/fl^* mice. There was no significant difference between cHET and *Gigyf1^fl/fl^* mice. Consistently, in LDB tests (Figure 3F), the cKO mice showed a significant increase in their preference for the dark box compared with *Gigyf1^fl/fl^* mice. This increase was not significant in cHET mice. These results indicate that *Gigyf1* haploinsufficiency in the developing brain has a very mild effect on anxiety-like behavior in mice. This is in contrast to homozygous KO *Gigyf1* mice, which showed considerably more anxiety-like behaviors.

To test learning and memory problems in *Gigyf1* KO mice, we subjected the mice to the Morris water maze test (MWM test) to evaluate cognition in *Gigyf1* cHET and cKO mice. In MWM tests (Figure 3G), *Gigyf1* cKO mice showed a decreased latency to discover targets during training trials, as well as a decreased latency to reach the target region during the probe trial compared with the *Gigyf1^fl/fl^* mice. However, this difference was not observed in cHET mice. Considering that the lower weight in the cKO mice might affect performance in the MWM test, we subjected the mice to the novel object recognition test (NOR test). In NOR tests (Figure 3H), *Gigyf1* cKO mice also showed a decrease in the total time spent exploring the nonfamiliar object and the discrimination index. These results suggest that *Gigyf1* haploinsufficiency has no significant effect on learning and memory in mice, and that homozygous KO *Gigyf1* in mice leads to severe learning and memory problems.

### Gigyf1 deficiency leads to a reduction of the upper cortical layers in mice.

**Figure 4:**
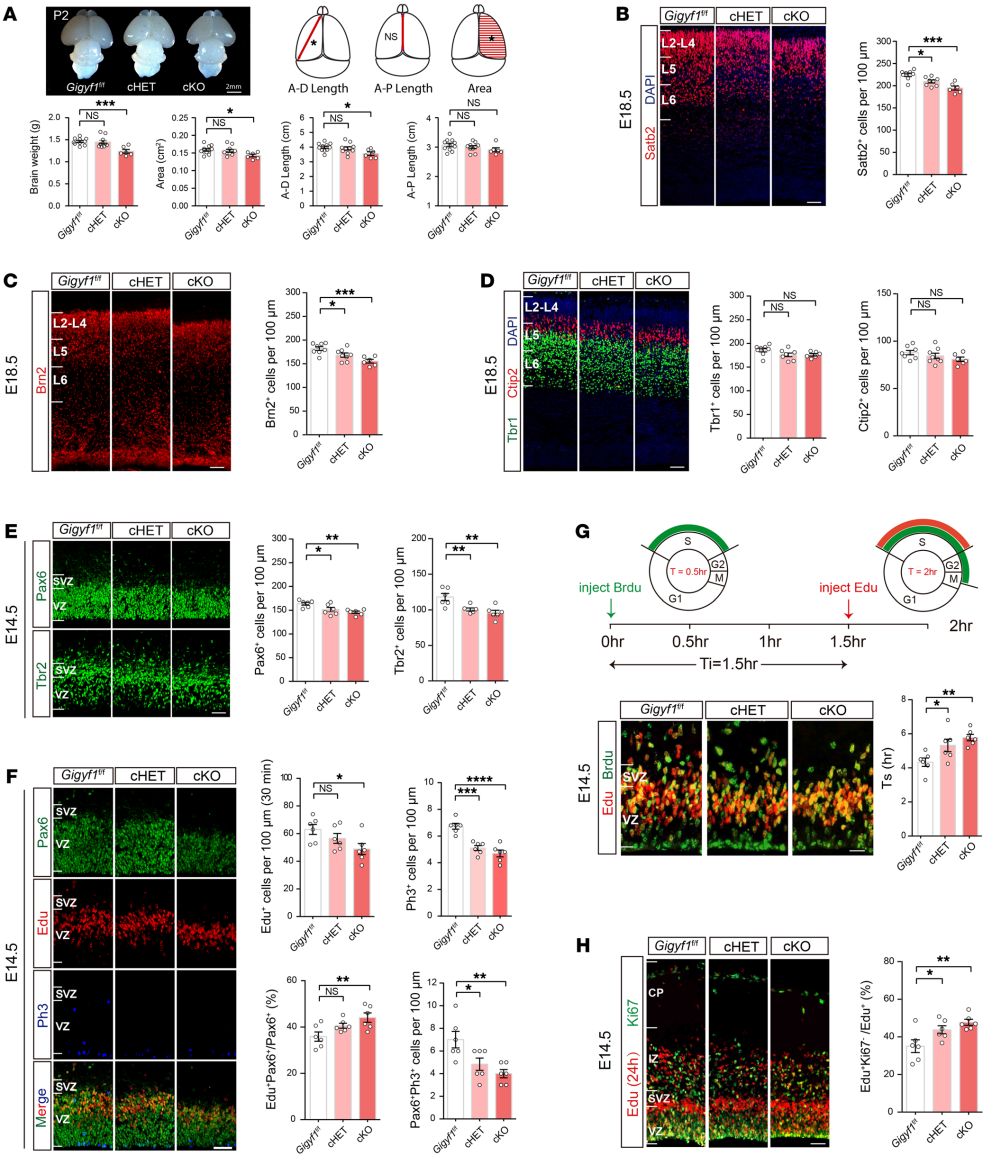
*Gigyf1* disruption in the developing brain disturbs neurogenesis. (**A**) Quantitative comparison of brain weight, cortical anterior-dorsal (A-D) length, anterior-posterior (A-P) length, and area in *Gigyf1^fl/fl^* (*n =* 11), cHET (*n =* 10), and cKO (*n =* 6) mice at P2. (**B**) Littermate cortices stained with Satb2 from *Gigyf1^fl/fl^* (*n =* 4), cHET (*n =* 4), and cKO (*n =* 3) mice at E18.5. Satb2^+^ cells per 100 μm of apical surface in L2–L4 were compared. (**C**) Littermate cortices stained with Brn2 from *Gigyf1^fl/fl^* (*n =* 4), cHET (*n =* 4), and cKO (*n =* 3) mice at E18.5. Brn2^+^ cells per 100 μm of apical surface in L2–L4 were compared. (**D**) Littermate cortices stained with Tbr1 and Ctip2 from *Gigyf1^fl/fl^* (*n =* 4), cHET (*n =* 4), and cKO (*n =* 3) mice at E18.5. Ctip2^+^ cells and Tbr1^+^ cells per 100 μm in L5 and L6 were compared. (**E**) Comparison of Pax6^+^ RGC (VZ) and Tbr2^+^ IPC (SVZ) populations per 100 μm of apical surface in *Gigyf1^fl/fl^* (*n =* 3), cHET (*n =* 3), and cKO (*n =* 3) mice at E14.5. (**F**) Comparison of Edu^+^ (SVZ) population, Ph3^+^ and Pax6^+^Ph3^+^ (VZ) population, Pax6^+^Edu^+^/Pax6^+^ proportion of apical surface from *Gigyf1^fl/fl^* (*n =* 3), cHET (*n =* 3), and cKO (*n =* 3) mice at E14.5. (**G**) Ki67 and Edu antibodies after 24 hour Edu pulse at E13.5. All cells that exited the cell cycles (Edu^+^/Ki 67^–^) were counted. The percentage of total Edu^+^ cells evaluated 24 hours after injection was analyzed. (**H**) S phase sequential labeling analysis of NPCs. EdU-Brdu double-stained cortical sections at E14.5 are shown. S phase durations were calculated (Ts=Ti/(Lcells/Scells)) and compared. All statistics were performed by 1-way ANOVA. Scale bars represent 50 μm. All data are represented as mean ± SEM. *P < 0.05, **P < 0.01, ***P < 0.001, ****P < 0.0001.

Growing evidence indicates that disrupted neocortical neurogenesis contributes substantially to ASD pathogenesis (13, 14). Using the transcriptome data from BrainSpan (https://www.brainspan.org/), we revealed that GIGYF1 was expressed broadly in human prenatal and postnatal brains, though slightly higher in the prenatal stage (Supplemental Figure 7A). By Western blotting, we revealed a similar pattern of Gigyf1 expression in the mouse cortex. Gigyf1 was highly expressed in the mouse prenatal cortex compared with the postnatal cortex, especially at E14.5 and E18.5 (Supplemental Figure 7B). To investigate the role of *Gigyf1* in embryonic cortical development, we first evaluated the overall brain size and cortical cytoarchitecture. We found that the *Gigyf1* cKO mice at P2 showed a mild decrease in cortical area and cortical length when compared with *Gigyf1^fl/fl^* mice (Figure 4A). No significant changes were observed in cHET mice. To explore whether *Gigyf1* deficiency changes the number of neurons in different cortical layers, we performed immunohistochemistry of layer-specific markers in the neocortex at E18.5. We detected fewer upper-layer neurons labeled with markers Satb2 and Brn2 in both cHET and cKO mice compared with the *Gigyf1^fl/fl^* mouse cortex (Figure 4, B and C). We did not observe a significant difference in the numbers of deeper-layer neurons including Tbr1^+^ and Ctip2^+^ cells in cHET and cKO mouse cortexes (Figure 4D). These data indicate that *Gigyf1* deficiency leads to a reduction in the number of upper-cortical neurons.

### Gigyf1 deficiency leads to decreased NPC proliferation.

The abnormalities of the cerebral cortex are thought to be due to the proliferation, differentiation, or migration of NPCs (15, 16). To explore the role of *Gigyf1* in NPC development, we first examined the numbers of Pax6^+^ radial glial cells (RGCs) and Tbr2^+^ intermediate progenitor cells (IPCs) in the cHET and cKO cortex at E14.5. We observed that both Pax6^+^ RGCs and Tbr2^+^ IPCs were decreased (Figure 4E), indicating that *Gigyf1* deficiency decreases the NPC pool.

To confirm that *Gigyf1* is indispensable for NPC proliferation and/or migration, we performed pulse-labeling experiments with Edu at E14.5 and analyzed brains at either 30 minutes or 4 days after pulse-labeling with Edu. We found that the migration of cHET and cKO cells remained unchanged at E18.5 (Supplemental Figure 8). We observed that Edu^+^ cells were decreased in both the cHET and cKO cortex at E14.5 (Figure 4F). However, the Pax6^+^Edu^+^/Pax6^+^ cells were increased in the cKO cortex (Figure 4F), indicating that *Gigyf1* deficiency may lead NPCs to stay in S phase and thus decrease their proliferation. To further determine whether the decreased proliferation of NPCs is responsible for the decreased NPC pool, we labeled Ph3 for immunohistochemistry analysis. We observed that Ph3^+^ cells and Ph3^+^Pax6^+^ cells were both decreased in the cHET and cKO cortex (Figure 4F), which is consistent with the reduction in the number of NPCs. To further validate that *Gigyf1* deficiency leads to a prolonged S phase in NPCs, we labeled S phase NPCs by giving pregnant dams sequential Brdu and Edu pulses separated by a 1.5-hour interval at E14.5; we then analyzed Brdu and Edu single- and double-labeled NPCs by immunohistochemistry to deduce the duration of S phase (T_S_) (Figure 4G). We found that the T_S_ of cHET and cKO NPCs was significantly longer than that of the *Gigyf1^fl/fl^* NPCs (Figure 4G). Similarly, increases in T_S_ were also observed in cHET and cKO embryos at E12.5 (Supplemental Figure 9).

To explore whether Gigyf1 has a functional role in NPC differentiation, we performed pulse-labeling experiments with Edu at E14.5 and analyzed brains after 24 hours. We found that the differentiation of NPCs in the cHET and cKO cortexes were accelerated compared with that in the *Gigyf1^fl/fl^* cortex (Figure 4H). Taken together, these results suggest that loss of *Gigyf1* in early brain development leads to a reduction in the number of upper-layer neurons, possibly linked to perturbations of proliferation and differentiation dynamics during cortical neurogenesis.

### GIGYF1 deletion interferes with the IGF-1R/ERK signaling pathway.

**Figure 5:**
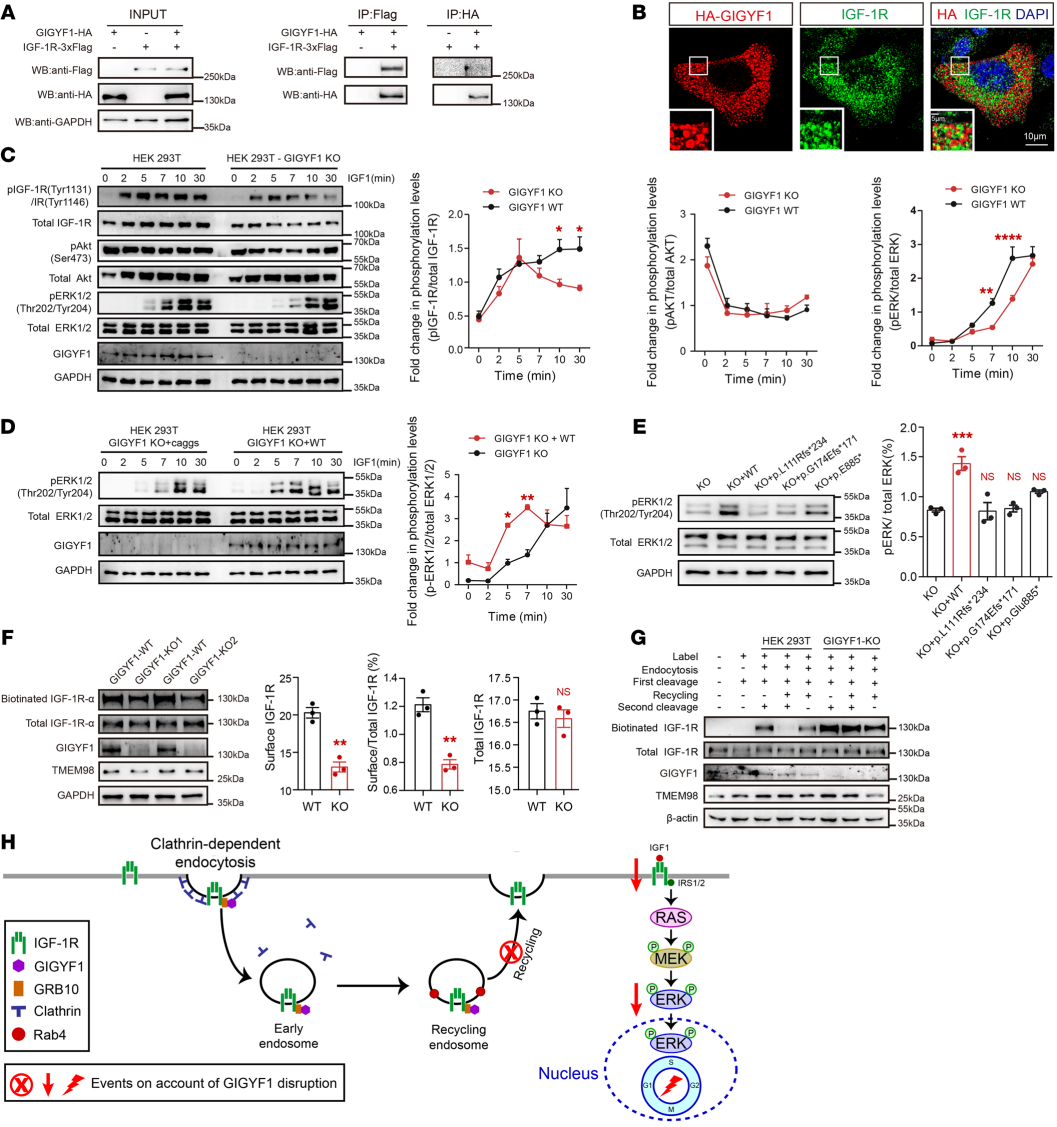
*GIGYF1* KO disrupts IGF-1R/ERK pathway by regulation of IGF-1R recycling. (**A**) Coimmunoprecipitation assay for GIGYF1 and IGF-1R in HEK293T cells. (**B**) Double immunofluorescence of GIGYF1 and IGF-1R in HeLa cells. Scale bars: 10 μm. Inset scale bars: 5 μm. (**C**) Immunoblots of the whole cell lysate showing levels of pIGF-1R, IGF-1R, pERK1/2, ERK1/2, pAkt, and Akt at different duration of IGF-1 stimulation. Statistical data were analyzed using 2-way ANOVA. (**D**) Immunoblots of the whole-cell lysates showing levels of pERK1/2, ERK1/2 in HEK293T *GIGYF1* KO cells expressing mock empty vector (pCAGGS-IRES-GFP) or HA-GIGYF1. The relative levels of pERK1/2 to ERK1/2 were quantified by densitometry and analyzed using 2-way ANOVA. (**E**) Immunoblot of pERK1/2 and ERK1/2 in the whole-cell lysates at 7 minutes of IGF-1 stimulation. Statistic data were analyzed using 1-way ANOVA. (**F**) Immunoblots of biotin-labelled IGF-1R, total IGF-1R and TMEM98. Total IGF-1R-α levels of unbiotinylated cells were determined. The protein levels of surface IGF-1R, surface IGF-1R/total IGF-1R, and total IGF-1R were quantified by densitometry from 3 biological replicates. Statistical data were analyzed using 2-tailed Student’s *t* tests. (**G**) Immunoblots of biotin-labeled IGF-1R and total IGF-1R at different conditions in a surface biotinylation recycling assay. *GIGYF1* KO HEK293T cells and control HEK293T cells (lanes 2–8) were surface labeled with sulfo-NHS-S-S-biotin. Cells (lanes 3–8) were incubated to endocytosis. The remaining surface biotin was cleaved with glutathione cleavage buffer (lanes 2–8). Cells were incubated for a second time to recycle (lanes 4–5 and 7–8), then the surface biotin was stripped for a second time (lanes 3–4 and 6–7). Lanes 5 and 8 were incubated to recycle without a second cleavage. The stage of biotinylation recycling assay for each lysate is indicated with + and –. (**H**) Working model of GIGYF1 regulation of IGF-1R/ERK pathway. All data are represented as mean ± SEM. *P < 0.05, **P < 0.01, ***P < 0.001, ****P < 0.0001. See complete unedited blots in the supplemental material.

The above data indicate that GIGYF1 plays a critical role in NPC proliferation and neurogenesis and that its mutation dysregulates the cell cycle during NPC development. Therefore, we further explored the potential underlying molecular mechanisms of GIGYF1 regulation of NPC development. Previous studies have shown that the GYF domain of GIGYF1 binds to Grb10, an adapter protein that, in turn, binds to IGF-1R (17). A GIGYF1 fragment containing the GYF domain interacts with IGF-1R at 2 minutes after IGF-1 stimulation (4). To further characterize the interaction of full-length GIGYF1 with IGF-1R, we performed a series of coimmunoprecipitation experiments. We found that Flag-tagged IGF-1R interacted with HA-tagged GIGYF1 (Figure 5A). Meanwhile, purified HA-tagged GIGYF1 interacted with Flag-tagged IGF-1R. To further validate the interaction between GIGYF1 and IGF-1R, we performed double immunofluorescence experiments and found that GIGYF1 colocalized with IGF-1R and GRB10 (Figure 5B and Supplemental Figure 10). These results are consistent with the previous implication that GIGYF1 forms a complex with IGF-1R and GRB10 (4).

IGF-1R is a plasma transmembrane receptor that is activated by IGF-1, which, in turn, activates the downstream ERK and AKT/mTOR pathways (18). Previous knockdown and overexpression studies support both negative and positive regulatory effects of GIGYF1 or its GYF domain on IGF-1R signaling, thus leaving its physiological role on IGF-1R signaling unclear (4, 19). To further clarify the function of GIGYF1 in IGF-1R signaling, we constructed GIGYF1 KO cell lines using HEK293T cells to determine whether *GIGYF1* deficiency is involved in IGF-1R induced signaling. We stimulated cells with IGF-1 at 0, 2, 5, 7, 10, and 30 minutes, and then assessed for phosphorylation of IGF-1R (pIGF-1R), pERK, and pAKT. We found that the ratio of pIGF-1R to IGF-1R at 10 and 30 minutes of IGF-1 stimulation was significantly decreased in *GIGYF1* KO cells compared with control cells (Figure 5C). Consistently, pERK/ERK at 7 and 10 minutes of IGF-1 stimulation was also decreased. Although we observed a slight decrease in pAKT at 0, 2, and 5 minutes of IGF-1 stimulation, this difference was not significant. To determine whether haploinsufficiency of *GIGYF1* also interferes with the IGF-1R/ERK pathway, we stimulated cells with IGF-1 at 0, 5, and 7 minutes in *GIGYF1* heterozygous cells and then assessed for phosphorylation of pERK. We found that pERK/ERK at 7 minutes of IGF-1 stimulation was also significantly decreased (Supplemental Figure 11). These results support that haploinsufficiency and deletion of GIGYF1 decrease pIGF-1R/pERK signaling.

To further elucidate whether GIGYF1 was involved in the regulation of the ERK pathway, we performed a rescue experiment by expressing WT plasmids of GIGYF1 in GIGYF1 KO cells under IGF-1 stimulation for 0, 2, 5, 7, 10, and 30 minutes (Figure 5D). WT plasmids of GIGYF1 significantly increased the pERK level after 5 and 7 minutes of IGF-1 stimulation. To explore whether disorder-related variants interfere with the ERK pathway, we performed a second rescue experiment using the recurrent *GIGYF1* LGD variant (p.L111Rfs*234) and another 2 LGD variants identified from our in-house cases (p.G174Efs*171 and p.E885*—1 from the N-terminus and 1 from the C-terminus, Supplemental Figure 2) after 7 minutes of IGF-1 stimulation. We found that all 3 *GIGYF1* LGD variants failed to rescue the decreased pERK level (Figure 5E). These data further validate that *GIGYF1* KO and disorder-related LGD variants perturb the IGF-1R/ERK signaling pathway.

### GIGYF1 regulates IGF-1R recycling.

The above data show that GIGYF1 deficiency suppresses the IGF-1R/ERK signaling pathway. However, how GIGYF1 regulates IGF-1R activation is unknown. The above data show that GIGYF1 mainly localizes to the cytoplasm in the form of vesicles (Figure 1D and Figure 5B) reminiscent of endosomes. It is also known that cell-surface IGF-1R traffics through endosomal vesicles (20). Once internalized, IGF-1R is first trafficked to early endosomes and subsequently transported either to late endosomes for degradation or to recycling endosomes for recycling (21) (Supplemental Figure 12). Based on this, we hypothesized that GIGYF1 might mediate the internalization and trafficking of IGF-1R. To test our hypothesis, we conducted double immunofluorescence experiments using antibodies for specific endosome markers. Intriguingly, we observed that HA-tagged GIGYF1 strongly colocalizes with the recycling endosomal marker Rab4 (Supplemental Figure 13). We also observed that HA-tagged GIGYF1 partially colocalizes with the coated vesicle marker clathrin and early endosomal markers EEA1 and Rab5A (Supplemental Figure 13). However, HA-tagged GIGYF1 does not colocalize with lysosomes (Lamp1) and other endosomal markers, including Rab7 and Rab11 (Supplemental Figure 14). These results indicate that GIGYF1 might be involved in clathrin-mediated endocytosis and Rab4-mediated recycling of internalized IGF-1R.

To further explore whether GIGYF1 regulates the recycling of IGF-1R, we conducted a surface biotinylation assay to detect surface expression of IGF-1R in *GIGYF1* KO cells. Cell-surface IGF-1R levels were significantly lower in *GIGYF1* KO compared with control cells (Figure 5F), but the total IGF-1R levels were not changed. In addition, the expression of TMEM98 — an IGF-1R–unrelated cell surface protein — remained unchanged, suggesting that GIGYF1 specifically affects IGF-1R. We next performed a surface biotinylation recycling assay (Supplemental Figure 15) to explore the effect of *GIGYF1* KO on IGF-1R expression in the cytoplasm. Interestingly, we found that KO of *GIGYF1* significantly increased the expression of IGF-1R in the cytoplasm, but the expression of IGF-1R was almost never detected in the control cells (Figure 5G, lane 4 versus lane 7). Taken together, these data demonstrate that GIGYF1 regulates the recycling of IGF-1R to the plasma membrane. Deficiency of *GIGYF1* led to decreased expression of IGF-1R in the plasma membrane (Figure 5H).

### Disturbance of IGF-1R/ERK signaling in early brain development of Gigyf1-deficient mice.

**Figure 6:**
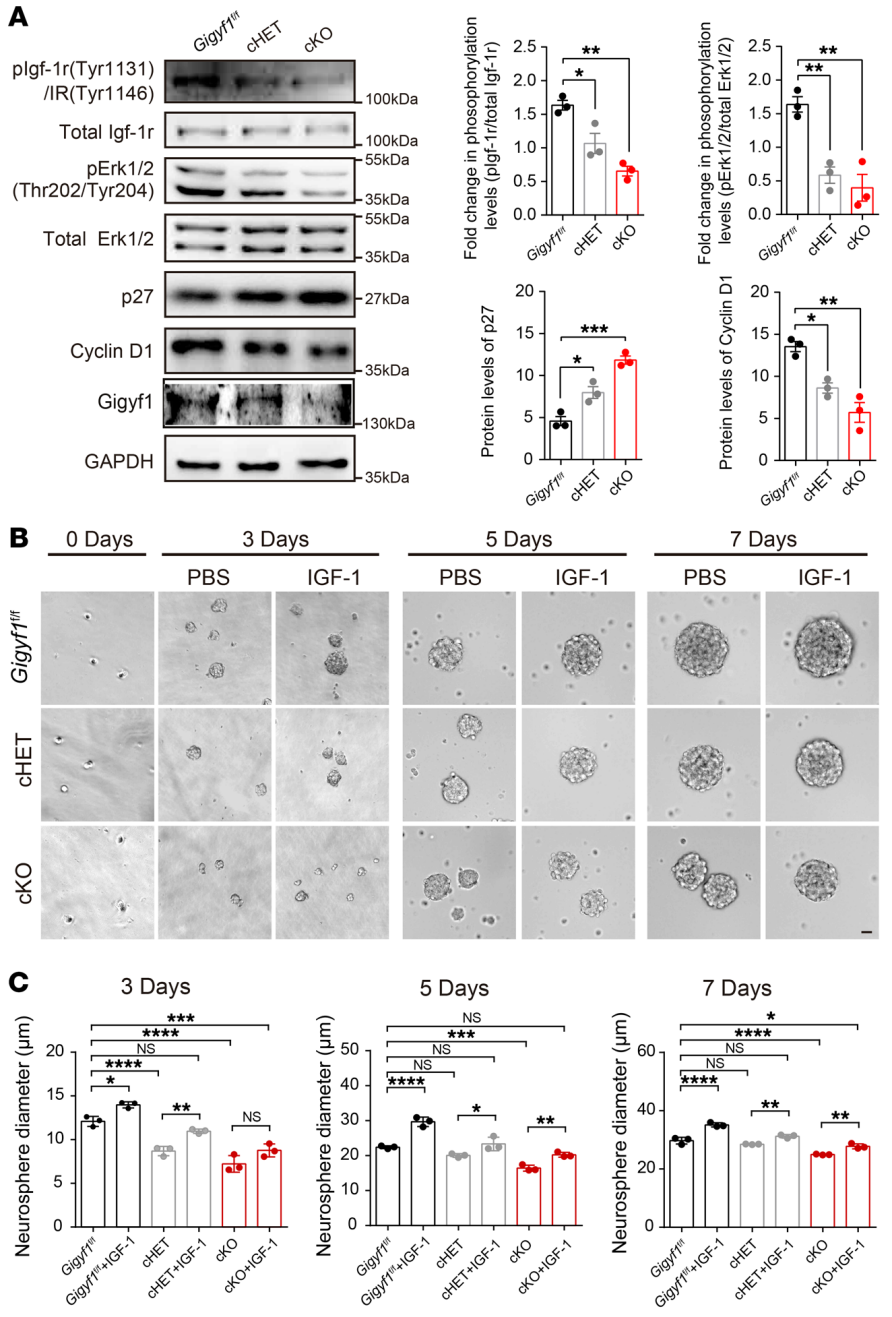
Dysregulation of IGF-1R/ERK signaling in *Gigyf1* deficiency mice. (**A**) Immunoblots of pIgf-1r, Igf-1r, pErk1/2, Erk1/2, p27, cyclin D1, and Gigyf1 in lysates from brain cortical tissue of *Gigyf1^fl/fl^*, cHET and cKO mice at E14.5. The relative levels of pIgf-1r/total Igf-1r, pErk-1r/total Erk, p27, and cyclin D1 were quantified by densitometry and compared using 1-way ANOVA. (**B**) Neurosphere formation assay. Neural progenitor cells are derived from *Gigyf1^fl/fl^*, cHET and cKO embryos. Representative images of *Gigyf1^fl/fl^*, cHET and cKO neurosphere are shown. Scale bar: 10 μm. (**C**) The diameters of *Gigyf1^fl/fl^*, cHET, and cKO neurospheres were calculated and compared. Experiments were performed for 3 trials and the statistics are based on the average of each condition from different trials. Statistical data are analyzed using 1-way ANOVA and 2-tailed *t* test. All data are represented as mean ± SEM. *P < 0.05, **P < 0.01, ***P < 0.001, ****P < 0.0001.

To validate whether GIGYF1 interferes with the IGF-1R/ERK signaling pathway in vivo, we performed immunoblotting experiments to detect pIgf-1r and pErk in *Gigyf1* KO mice at E14.5. We observed that pIgf-1r and pErk levels were significantly diminished in the cortical lysates of both cHET and cKO mice compared with *Gigyf1^fl/fl^* mice (Figure 6A). Consistently, immunohistochemistry of pErk revealed decreased fluorescence intensity of pErk in the cortex of cHET and cKO mice (Supplemental Figure 16). ERK activation plays a fundamental role in G1/S transition (22). In cell cycle regulation, ERK activity regulates the induction of cyclin D1 and the downregulation of p27 (23). Since we observed disturbed NPC cell cycle dynamics in *Gigyf1*-deficient mice, we next examined whether p27 and cyclin D1 were also dysregulated. We found significantly increased expression of p27 and decreased expression of cyclin D1 (Figure 6A). These data together indicate that altered cell cycle dynamics of NPCs are most likely the result of disruption of the IGF-1R/ERK signaling pathway due to the lack of *Gigyf1* in the mouse.

We then tested whether IGF-1 could rescue *GIGYF1*-related pathogenesis. We adopted neurosphere formation assays at E14.5. We recorded and measured the size of neurospheres at 0, 3, 5, and 7 days of culturing in vitro. We found that the diameters of cHET neurospheres were significantly smaller than those of *Gigyf1^fl/fl^* at 3 days, and that the diameters of cKO neurospheres were significantly smaller at all time points (Figure 6, B and C), indicating the impaired proliferation of NPCs in cHET and cKO neurospheres, which is consistent with the above observation of disruption of the IGF-1R/ERK signaling pathway in GIGYF1-deficient mice. In contrast, IGF-1 stimulation could enhanced the ability of both cHET and cKO neurospheres to proliferate (Figure 6, B and C). Specifically, IGF-1 stimulation could rescue the decreased diameter of cHET neurospheres at all time points and the decreased diameter of cKO neurospheres at 5 days. IGF-1 stimulation could partially rescue the decreased diameter of neurospheres in cKO at 3 days and 7 days. These data indicate that IGF-1 can ameliorate the proliferation impairments in *Gigyf1*-deficient mice.

## Discussion

In this study, we comprehensively characterized the mutation pattern and inheritance modes of *GIGYF1* mutations using large, recently sequenced ASD cohorts. Our data confirm de novo enrichment of *GIGYF1* LGD variants and highlight the important contribution of inherited LGD variants in potential ASD risk. Notably, we report a recurrent frameshift variant eliciting a loss-of-function effect on ERK signaling activity that accounts for 40% of individuals with ASD with GIGYF1 LGD variants and 0.064% of individuals with ASD. Our findings emphasize the importance of investigating rare, inherited, recurrent variants in ASD or NDD risk, even with a sample size with limited statistical power for this class of variants. The molecular basis for this recurrent deletion is unknown, but we noticed that it is located adjacent to a polyguanine stretch. In yeast experimental assays, such polyG tracts (albeit typically longer, at least 13) have been shown to increase mutability both locally and distally, possibly through error-prone translation synthesis and repair pathways (24).

Our phenotypic association analysis revealed that individuals with ASD with *GIGYF1* heterozygous LGD variants shared ASD core symptoms, but with a lower prevalence of cognitive impairment. Although *GIGYF1* LGD variants were also identified among family members without ASD diagnoses, we found evidence of ASD/NDD endophenotypes, such as language/motor developmental delay as well as social impairment. These findings suggest that *GIGYF1* may represent an important ASD risk gene underlying core ASD phenotypes without significant cognitive impairments. Indeed, our data show that the haploinsufficiency of *Gigyf1* in the developing mouse brain did not contribute to learning and memory deficits associated with cognitive impairments. In contrast, homozygous knockouts of *Gigyf1* in the developing brain caused more severe ASD core symptoms, anxiety-like behaviors, and severe cognitive problems, consistent with a dosage effect of loss-of-function *Gigyf1* mutations on phenotypic severity.

The functional roles of GIGYF1 in neurodevelopment have not been previously characterized. During the early stage of embryonic neurodevelopment, NPCs in the ventricular zone (VZ) and the subventricular zone (SVZ) generate the complex cytoarchitecture and the final adult cortex as a result of NPC proliferative or neurogenic divisions. The balance of this process determines normal cortex composition and function (15, 16). Using our nervous system conditional KO mouse model, we found that Gigyf1 was essential for neocortical neurogenesis, which is strongly implicated in ASD pathology (14). Our data revealed a reduced number of neurons of the upper but not deeper layers in *Gigyf1*-deficient mice. The explanation might be that the reduction of the ERK signaling pathway led to premature neurogenesis of NPCs in early stages, which compensates for the presumed reduced deeper-layer neurons (25). We proposed that *Gigyf1* deficiency alters the homeostasis of NPC proliferation and differentiation, which, in turn, affects the development of the cerebral cortex.

Regulation of the cell cycle by the IGF-1R/ERK pathway has been well characterized (26). Although GIGYF1 has been shown to be involved in the regulation of the IGF-1R signaling pathway, results have been contradictory (4, 19). In this study, we confirmed that *GIGYF1* deficiency reduced pIGF-1R and downregulated the downstream ERK signaling pathway. In addition, the *GIGYF1* LGD variants identified from individuals with ASD were unable to rescue the decreased pERK levels, further supporting the hypothesis that IGF-1R/ERK dysregulation underlies, in part, the pathology of *GIGYF1*-associated individuals with ASD. Importantly, in vivo analysis further supports the dysregulation of the Igf-1r/Erk signaling pathway in *Gigyf1*-deficient mice. Besides this reduction in the pIgf-1r/pErk signaling pathway, 2 critical cell cycle regulators, p27 and cyclin D1, were also perturbed in *Gigyf1*-deficient mice. We propose that the abnormal outcomes on neurogenesis and NPC proliferation were likely due to the disruption of the Igf-1r/Erk signaling pathway by loss of *Gigyf1*, although our study did not provide clear causal evidence for the disrupted Igf-1r/Erk signaling in cortical neurogenesis and behavioral phenotypes observed in the cHET and cKO mice. Whether this is the causal, or a causal, factor in the phenotypes remains to be determined.

We also investigated how GIGYF1 regulates the IGF-1R signaling pathway. It is known that once IGF-1R is internalized, it is recycled, degraded, or translocated to the intracellular membrane compartments of the Golgi apparatus or the nucleus (21). We report that GIGYF1 strongly colocalized with the recycling endosomal marker Rab4. Rab4 and Rab11 had both been proposed to be involved in the “fast” and “slow” recycling pathways, respectively (27). We did not detect colocalization of GIGYF1 with Rab11, indicating that GIGYF1 predominantly mediated the fast recycling pathway. Our surface biotinylation assay further confirmed that GIGYF1 regulated the recycling of GIGYF1 to the cell surface. We believe that these results suggest GIGYF1 as a novel regulator of IGF-1R recycling, which might be an important target for elucidating the recycling mechanisms specific for IGF-1R.

In summary, our study demonstrated that haploinsufficiency of *GIGYF1* in humans and mice led to core autistic behaviors with less-significant cognitive impairments. We propose that disruption of *GIGYF1*, which we believe to be a new regulator of IGF-1R fast recycling, leads to inactivation of the IGF-1R/ERK signaling pathway contributing to neurodevelopment and autistic behaviors in mice through perturbation of normal the cell cycle dynamics of NPCs early in brain development. The discovery of inherited and de novo *GIGYF1* pathogenic variants in individuals with ASD could enhance genetic diagnosis and studies of transmission within families, which is critical for genetic counseling, especially among multiplex families. The mouse model and molecular insights further highlight the importance of the IGF-1R/ERK pathway in the molecular pathogenesis of ASD.

## Methods

### WES and WGS data.

The WES or WGS data of 7 sub-cohorts of from SPARK (8) were included in this study, including SPARK_pilot, SPARK_WES1, SPARK_WES2, SPARK_WES3, SPARK_WGS1, SPARK_WGS2, and SPARK_WGS3. After removing the duplicates in the 7 sub-cohorts, 68,560 individuals, including 33,241 individuals with ASD with WES/WGS data passing quality control, were included in our study. The WGS data of 2,337 trio or simplex quad families from the SSC data set (9) were used in this study. VCF files of SPARK_pilot, SPARK_WES1, SPARK_WES2, SPARK_WES3, SPARK_WGS1, SPARK_WGS2, and SPARK_WGS3 were downloaded from SFARI Base (https://base.sfari.org/). BCFtools (28), VCFtools (29) and GATK (30) (v4.1.8.1) were applied to left-align, normalize, extract and filter rare LGD events (allele frequency < 0.1% in gnomAD v2.1.1 nonneuro subset exomes (31) in the VCF files. We applied genotype quality (GQ), read-depth (RD) and allele balance (AB) filters for QC and variants filtering. LGD variants within 0.25 < AB < 0.75, DP > 10 and GQ > 25 were retained. All *GIGYF1* LGD mutations were visualized using Integrative Genomics Viewer (32) in order to get rid of likely variant calling artifacts. ANNOVAR (33) was used to annotate the variants.

### De novo enrichment and transmission disequilibrium analysis.

Excess of *GIGYF1* de novo LGD variants was analyzed using 2 probabilistic models: denovolyzeR (34) and CH model (35). Briefly, we derived the expected number of de novo LGD events in a given population based on the mutability of *GIGYF1* and the number of probands sequenced. We then compared the observed number of de novo LGD variants against expectation using a Poisson framework (denovolyzeR) or binomial model (CH model). We applied the rare-variant transmission disequlibrium test (RV-TDT) (36) for transmission disequilibrium analysis of *GIGYF1* LGD variants in 49 families with data from at least 1 parent available.

### Clinical data and analysis.

For the SPARK cohort, clinical data used in this study were pulled from SPARK_Collection_Version6. SCQ total score, RBS-R total score, and cognitive impairment conditions data were extracted from the core_descriptive_variables.csv file. The clinical records of DD (global developmental delay, speech and language delay, learning disability, motor delay, or developmental coordination disorder), behavior problems (attention deficit-hyperactivity disorder (ADHD) or attention deficit disorder (ADD), conduct disorder, intermittent explosive disorder, oppositional defiant disorder), and neuropsychiatric problems (anxiety disorder, bipolar disorder, depression or dysthymia, disruptive mood dysregulation disorder, hoarding, obsessive-compulsive disorder, separation anxiety, social anxiety disorder/social phobia, seizure disorder or epilepsy, personality disorder, schizophrenia, other psychosis or schizoaffective disorder, Tourette syndrome, or tic disorder) were extracted from the basic_medical_screening.csv file. For the SSC cohort, phenotype information is available from the SSC version 15.3 phenotype data set. SCQ total score and SRS t score were derived from the srs_parent.csv and scq_life.csv files, respectively. Comparisons between groups were performed using Mann-Whitney U test (qualitative data) or Fisher’s exact test (quantitative data). Down sampling analysis was performed by permutation test (random sampling 1,000,000 times).

### Mice.

The *Gigyf1* conditional KO mice were generated in GemPharmatech, Co., Ltd. Mice were housed in a constant-temperature and humidity environment with relatively stable conditions (generally 22°C–24°C, 70% humidity, 12 hours of light and 12 hours of darkness) and allowed to eat and drink freely. The genetic background of all mice used in this study is C56BL/6J. Age- and sex-matched littermate pairs were used in the experiments.

### Behavioral tests.

All behavioral tests were performed using age-matched male littermates. The age of mice used for the first behavioral test was around 4 weeks. The age range of mice used for behavioral tests was 4–8 weeks. Mice had at least 24 hours of rest time between tests. All experimental data were analyzed using AVTAS version 5.0 single tracking, Animal Video Tracking Analysis System (AniLab Software and Instruments, guoj). All tests were conducted in a blind manner. Detailed methods for specific behavioral tests (3-chamber test, marble burying test, digging, rearing, and grooming tests, MWM test, LDB test, EPM test, OF test, NOR test) can be found in Supplemental Methods.

### Immunohistology.

Immunohistology was performed on 20 μm frozen tissue sections. Brain slices used local tissue structure for spatial matching to ensure accurate spatial comparisons. The prepared brain slices were baked at 60°C for 2 hours. Frozen sections were washed with DPBS for 10 minutes, and, for BrdU staining, sections were treated with 2 M HCl at 37°C for 30 minutes and 0.1 M sodium borate buffer, pH 8.5, for 10 minutes at room temperature. They were then blocked with 5% BSA in 0.1% PBST for 1 hour at room temperature. Sections were incubated overnight at 4°C with primary antibodies (rabbit Tbr1; rabbit 488; rabbit Cy3; rabbit Pax6; rabbit Tbr2; rat BrdU; rabbit Ctip2; mouse Ph3; mouse Brn2). After washing 3 times with DPBS, secondary antibodies were applied to sections for 1 hour at room temperature. Sections were stained with DAPI for 1 minute and covered with a cover glass. Fluorescence images were acquired by Zeiss LSM 880 confocal microscope and analyzed in ImageJ software.

### Injection of S-phase tracer.

BrdU/EdU double-labeling was carried out according to Houlihan et al. (37). Briefly, pregnant females were injected intraperitoneally with BrdU, (Millipore-Sigma) (50 mg/kg body weight) and 1.5 hours later with the same dose of 5-Ethynyl-2’- deoxyuridine (EdU, Millipore-Sigma) and sacrificed after 0.5 hours. For EdU single-labeling, pregnant mice were injected intraperitoneally with EdU (50 mg/kg body weight) at E14.5 and sacrificed after 0.5 hours, 24 hours, or 4 days. The obtained mouse brain slices were stained for Edu immunohistochemistry using the Click-iT Edu kit (C10338,Thermo Fisher Scientific).

### Plasmids.

Full-length *GIGYF1*, purchased from Youbio Biological Technology Company, was cloned into the pCAGGS-IRES-GFP vector using SgsI and XhoI restriction sites. LGD mutations of *GIGYF1* (p.L111Rfs*234, p.G174Efs*171, p.E885*) were generated by site-directed mutagenesis using Phanta Max Super-Fidelity DNA Polymerase (P505-d1,Vazyme). Plasmids of pcDNA3.1-3xFlag-IGF-1R were purchased from Youbio Biological Technology Company. All constructs were confirmed by Sanger sequencing.

### Cell culture and transfection.

HEK293T cells (CBP60439, Cobioer) and HeLa cells (CBP60232, Cobioer) were cultured in DMEM medium with 10% FBS (10100147, Thermo Fisher Scientific), 1% penicillin and streptomycin (15140122, Thermo Fisher Scientific). All cells were cultured at 37°C in a humidified incubator with 5% CO2. Lipofectamine 3000 reagent (L3000015, Thermo Fisher Scientific) was used for transfection, following the manufacturer’s protocol.

### LentiCRISPR V2-mediated GIGYF1 KO cells.

SgRNA of GIGYF1 was designed through Sequence Scan for CRISPR (http://crispr.dfci.harvard.edu/SSC), and synthesized DNA oligos were inserted into LentiCRISPR V2 vectors (52961, Addgene plasmid) using FastDigest BsmBI (FD0454, Thermo Fisher Scientific). The constructs were confirmed by Sanger sequencing. HEK293T cells were transiently transfected with sgRNA, pCMV-VSV-G (8454, Addgene plasmid) and psPAX2 (12260, Addgene plasmid) for 2 days, then concentrated and precipitated through ultrafiltration. Lentiviruses infected HEK293T cells for 2 days followed by the addition of 2 μg/mL puromycin for 3 days. Cells were diluted to 96-well plates for monoclonal cell selection, and successful knockouts were verified by Western blot using the GIGYF1 antibody from Bethyl.

### Antibodies.

The following primary antibodies were used: rabbit HA-tag (1:1,000, CST, 3724s), rabbit Flag-tag (1:1,000, CST, 14793s), rabbit Clathrin Heavy Chain (1:50, CST, 4796s), rabbit Caveolin-1 (1:200, CST, 3267s), rabbit EEA1 (1:200, CST, 3288s), mouse Rab5A (1:400, CST, 46449s), rabbit Rab4 (1:200, CST, 2167s), rabbit Rab11 (1:50, CST, 5589s), rabbit Rab7 (1:50, CST, 9367s), rabbit Lamp1 (1:100, CST, 9091s), rabbit phospho-IGF-1R-β (Tyr1131)/insulin receptor β (Tyr1146) (1:1,000, CST, 3021s), rabbit IGF-1R-β (1:1,000, CST, 9750s), rabbit phospho-Akt (Ser473) (1:1,000, CST, 4060s), rabbit Akt (1:1,000, CST, 9272s), phospho-p44/42 MAPK (Thr202/Tyr204) (1:1,000, CST, 4370s), rabbit p44/42 MAPK (1:1,000, CST, 9102s), rabbit p27 Kip1 (1:1,000, CST, 3686s), rabbit Cyclin D1 (1:1,000, CST, 55506s), rabbit Tbr1 (1:400, CST, 49661s), rabbit Ki67 (1:400, CST, 9129s), rabbit Grb10 (1:1,000, Santa Cruz, sc-74509), mouse IGF-1R-α (1:1,000, Santa Cruz, sc-81464), mouse Brn2 (1:100, Santa Cruz, sc-393324), rabbit Satb2 (1:500, Abcam, ab92446), rabbit Tbr2 (1:100, Abcam, ab23345), rat BrdU (1:500, Abcam, ab6326), rabbit Ctip2 (1:100, Abcam, ab18465), rabbit 488 (1:500, Thermo Fisher Scientific, SA5-10018), rabbit Cy3 (1:500, Thermo Fisher Scientific, A10522), chicken GFP (1:500, Aves Labs, GFP-1020), rabbit Pax6 (1:100, BioLegend, PRB-278P), rabbit GIGYF1 (1:1,000, Bethyl, A304-132A-M), mouse Ph3 (1:2,000, Millipore-Sigma, 07-424), and rabbit TMEM98 (1:1,000, Millipore-Sigma, HPA053385).

### Immunofluorescence assay.

HeLa cells were grown in 12-well plates to about 30%–50% confluence and were transiently transfected with 1.5 μg of expression plasmid. After 24 hours, cells were washed with PBS and fixed with 4% paraformaldehyde (V900894-100G, Sigma-Aldrich) for 10 minutes, and then blocked with 5% BSA (FA016-25G, Genview) in 0.1% PBST for 1 hour at room temperature. Cells were incubated overnight at 4°C with primary antibodies. After washing 3 times with PBS, cells were incubated with corresponding secondary antibodies for 1 hour at room temperature. Cells were stained with DAPI (D9542, Sigma-Aldrich) for 1 minute. The cells were then covered with a cover glass and then fluorescence was visualized by confocal microscopy (341-H, Leica).

### Western blot assay.

*GIGYF1* KO HEK293T cells and control HEK293T cells were stimulated with 200 μg/mL IGF1 (PHG0078, Thermo Fisher Scientific) at different times (0, 2, 5, 7, 10, or 30 minutes) and were lysed at 48 hours in 1% Triton X-100 lysis buffer (10 mM Tris-HCl pH 7.4, 5 mM EDTA, 1% Triton X-100, Halt protease and phosphatase inhibitor cocktail [78446, Thermo Fisher Scientific]).

For a rescue experiment, *GIGYF1* KO HEK293T cells were transiently transfected with 1.5 μg WT, p.L111Rfs*234, p.G174Efs*171, and p.E885* GIGYF1 plasmids. After 24 hours of transfection, the cells were stimulated with 200 μg/mL IGF1 (PHG0078, Thermo Fisher Scientific). After 7 minutes, cells were lysed in 1% Triton X-100 lysis buffer.

The cortex tissues were obtained from *Gigyf1^fl/fl^*, cHET, and cKO mice at E14.5, then were homogenized with 500 μL of 1% Triton X-100 lysis buffer. The homogenates were centrifuged at 10,000*g* for 15 minutes at 4°C.

All of the above supernatants were separated by SDS-PAGE and transferred from gels to polyvinylidene difluoride membranes (IPVH15150, Millipore-Sigma), blocked for an hour at room temperature, followed by incubation in primary antibody overnight at 4°C and secondary antibody incubation at 1:10,000. Protein-antibody complexes were detected with SuperSignal West Femto Maximum Sensitivity Substrate (A38556, Thermo Fisher Scientific).

### Immunoprecipitation assay.

pCAGGS-IRES-GFP-HA-GIGYF1 and pcDNA3.1-3xFlag-IGF-1R were cotransfected with 2 μg of each plasmid in HEK293T cells. After 24 hours, cells were lysed in NP40 lysis buffer (P0013F, Beyotime Biotechnology) and centrifuged at 8,000*g* for 5 minutes at 4°C. The supernatants were subjected to immunoprecipitation of anti-HA immunomagnetic beads (B26201, Bimake) and anti-Flag immunomagnetic beads (B23101, Bimake). The lysates (1–2 mg protein) were respectively incubated with 10 μL beads overnight at 4°C. After the beads were washed with PBST 3 times, proteins were eluted with SDS sample buffer (10 mM Tris, pH 7.8, 3% SDS, 5% glycerol and 0.02% bromophenol blue) and were detected by Western blotting.

### Surface biotinylation assay.

Surface biotinylation assay was carried out according to the protocal in Truong et al. (38). *GIGYF1* KO HEK293T cells and control HEK293T cells were plated into 6 cm dishes for 2 days. After discarding the medium, cells were washed twice with 2 mL PBS/CM (PBS containing 1 mM MgCl_2_ and 1.3 mM CaCl_2_), then incubated with a freshly made solution of SulfoNHS-SS-Biotin (PG82077, Thermo Fisher Scientific) (0.25 mg/mL) for 30 minutes at 4°C. 50 mM NH4Cl was added to PBS/CM to stop the reaction at 4°C for 10 minutes. The cells were lysed with lysis buffer (0.2% SDS, 1% Triton X-100, 0.5% deoxycholic acid, 50 mM Tris-HCl, pH 8.0, 150 mM NaCl, protease inhibitors), and then 500 μg of biotinylated proteins were purified with NeutrAvidin Agarose (29200, Thermo Fisher Scientific) for 1 hour at room temperature. The purified biotinylated proteins were incubated in elution buffer (50 mM DTT, 2% SDS, 62.5 mM Tris-HCl, pH 6.8, 10% glycerol) for 1 hour at room temperature to remove the biotin. The expression of surface IGF-1R was detected by Western blotting.

### Surface biotinylation recycling assay.

Surface biotinylation assay was also carried out according to the protocol in Truong et al. (38). *GIGYF1* KO and control HEK293T cells were surface labeled with Sulfo-NHS-SS-Biotin (0.25 mg/mL). The labelling reaction was quenched, and then cells were incubated with fresh medium at 37˚C for 30 minutes for endocytosis. Remaining surface biotin was cleaved with glutathione cleavage buffer (50 mM glutathione, 75 mM NaCl, 10 mM EDTA, 1% BSA, 75 mM NaOH) at 37˚C for 30 minutes, twice. To detect IGF-1R endocytosis, the cells were lysed and biotinylated proteins were purified with NeutrAvidin Agarose. Cells were incubated with serum-free growth medium for a second time at 37˚C for 30 minutes to recycle, and were surface stripped for a second time. Then cells were lysed and incubated with NeutrAvidin Agarose. The beads were washed, proteins were eluted using DTT and separated by SDS-PAGE. Proteins were immunoblotted by Western blotting.

### Neurosphere culture.

The cortical tissues of embryonic mice at E14.5 were isolated. The obtained cortical tissues were mechanically triturated into single cells with Accutase (00-4555-56, Thermo Fisher Scientific) and washed twice with DMEM plus 10% FBS medium. Cells were subsequently resuspended in neurosphere medium. Cells were plated at a cell density of 3 × 10^4^–10^5^ cells/mL on uncoated 6-well dishes and cultured in DMEM/F12 (11320033, Gibco), B27 supplement (17504044, Gibco), 10 ng/mL bFGF (Peprotech), and 20 ng/mL EGF (Invitrogen). For the self-renewal analysis of NPCs, primary neurospheres were dissociated with Accutase and passaged at a cell density of 100 cells/mL on uncoated 24-well dishes using the same culture conditions as in the primary culture. The size of primary neurospheres was counted after 3, 5, and 7 days of culture.

### Data and code availability.

The WES and WGS data used in this study are available from the following resources. The GATK VCF files for SPARK WES and WGS data and SPARK phenotype data used in this study are available through SFARI and available to approved researchers at SFARI Base (accession nos. SFARI_SPARK_WES_p, SFARI_SPARK_WES_1, SFARI_SPARK_WES_2, SFARI_SPARK_WES_3, SFARI_SPARK_WGS_1, SFARI_SPARK_WGS_2, SFARI_SPARK_WGS_3). All GATK VCF files for SSC WGS data and SSC phenotype data are available by request from SFARI Base (accession no. SFARI_SSC_WGS). All software used in this study is publicly available.

### Statistics.

All experiments were performed with a minimum of 3 independent replicates. Unpaired or paired Student’s *t* test, 1-way or 2-way ANOVA were performed, where appropriate, to analyze data. All data are presented with mean values ± SEM. *P* values of less than 0.05 were considered significant. Detailed statistical methods and results for Figure 3, 4, 5, and 6 and Supplemental Figures 6, 8, and 9 are available in Supplemental materials.

### Study approval.

Written informed consent was obtained from study participants or their parents or legal guardians, in line with local IRB requirements at the time of collection. The IRB of the Central South University approved this study (#2019-1-23). All animal experiments were complied with all relevant ethical regulations and were approved by the IRB of Central South University (IRB#2019-2-23). The analysis of SPARK data was approved by the IRB of Central South University (IRB #2019-1-17).

## Author contributions

HG, GC, BY, ST, JT, and KX designed and conceived this study. ST and HG analyzed and interpreted the genotype and phenotype data. GC and BY performed the mouse behavioral and neurogenesis analyses. BY, GC, and JT designed the cell biology, histology, and biochemistry studies and analyzed the data. EEE, JT, XJ, QZ, XZ, QJ, Y Hua, Y Han, SL, QP, ZH, and LY helped with data interpretation. HG and KX supervised the work. GC, BY, ST, HG, JT, EEE, and KX wrote and revised the manuscript. Other authors including KH, RC, JS, IC, AFJ, SA, SDK, RAB, RKE, ECKN, LLPR, MLSFS, DMM, DB, JDS, SMK, and MJI, contributed and interpreted the genetic and clinical data recruited from an international collaborative network. All authors commented on the manuscript and approved the final manuscript. GC, BY, ST, and JT made equally important contributions from 4 different angles of this study and are thus considered co–first authors. The order of first authorship was determined by the volume of work each author contributed to the study.

## Supplementary Material

Supplemental data

Supplemental data set 1

Supplemental tables 1-9

## Version 1 08/02/2022

In-Press Preview

## Version 2 10/03/2022

Electronic publication
