# Supplementary material for: GIGYF1 disruption associates with autism and impaired IGF-1R signaling
Source: J Clin Invest. 2022 Oct 3;132(19):e159806. doi: 10.1172/JCI159806 (PMC9525121; doi:10.1172/JCI159806)
Supplement: Supplemental data [file jci-132-159806-s113.pdf]

Supplementary Figures and Methods

Supplementary Figures

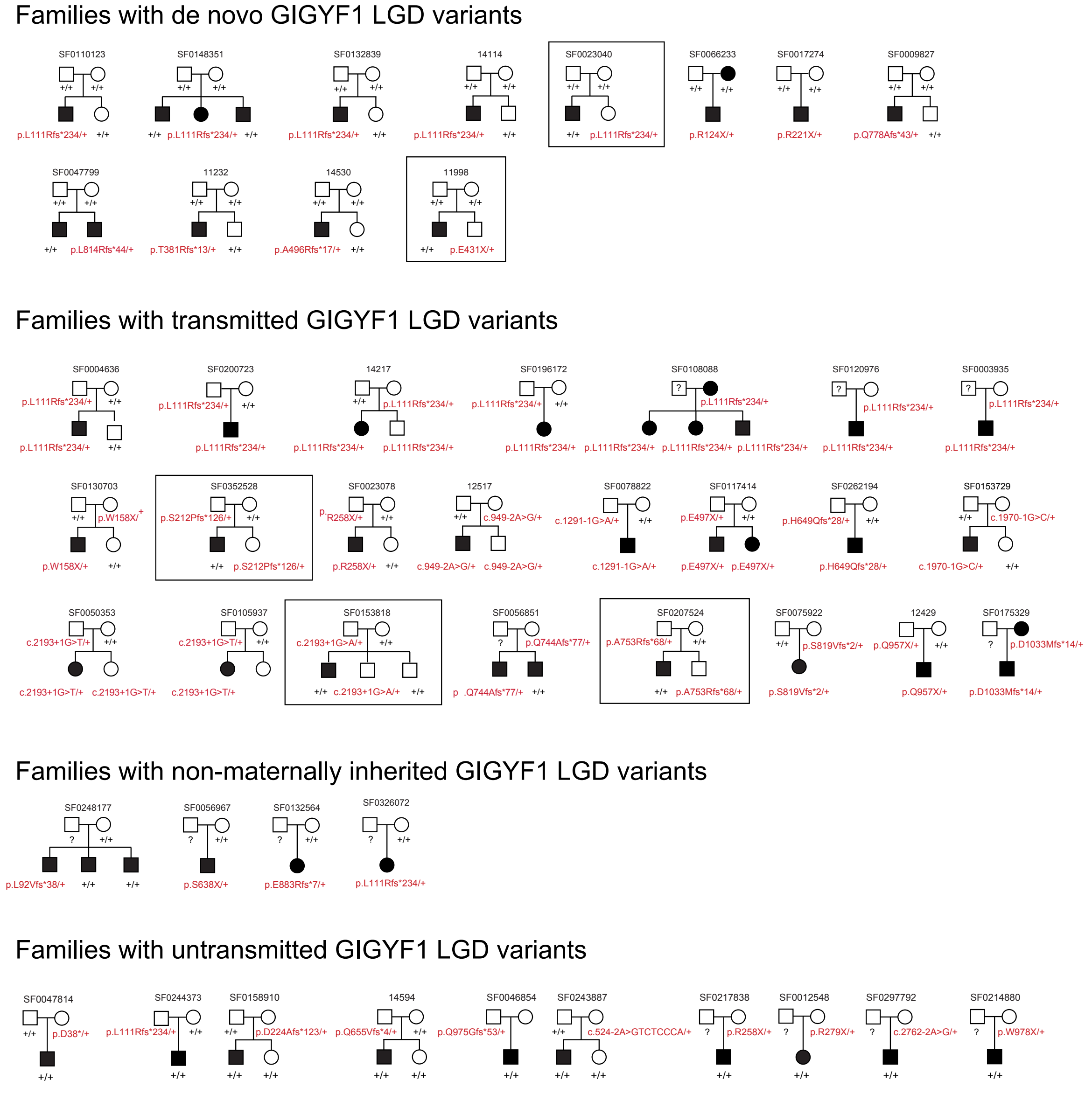

Supplemental Figure 1. All pedigree plots of biological parent(s) available families with *GIGYF1* LGD variants from SPARK and SSC. Families with *de novo GIGYF1* LGD variant in unaffected siblings or with transmitted *GIGYF1* LGD variant only to unaffected siblings are squared. Black filled circles or squares represent individuals with ASD diagnosis.

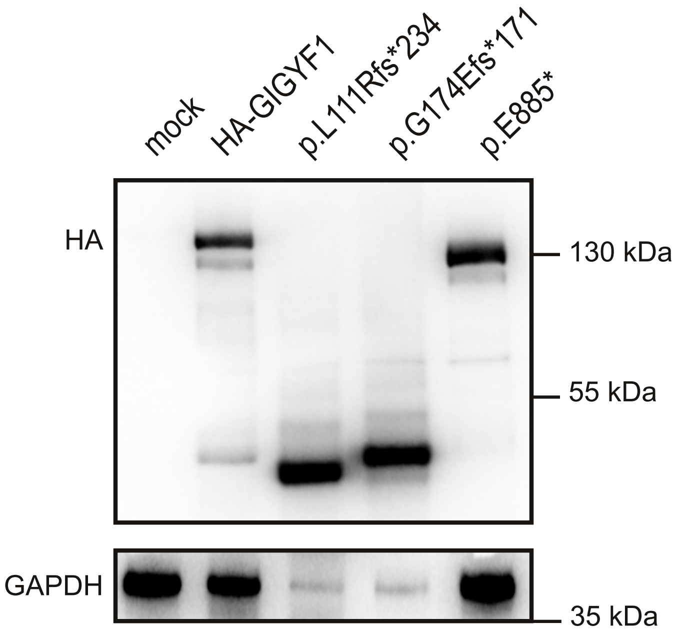

Supplemental Figure 2. Immunoblots of HA in the whole-cell lysates from HEK293T expressing GIGYF-WT, pCAGGS-IRES-GFP, GIGYF-p.L111Rfe*234, GIGYF-p.G174Efs*171 and GIGYF-p.E885*. The sample loading quantity of p.L111Rfe*234 and p.G174Efs*171 are 1/5 compared to other lanes.

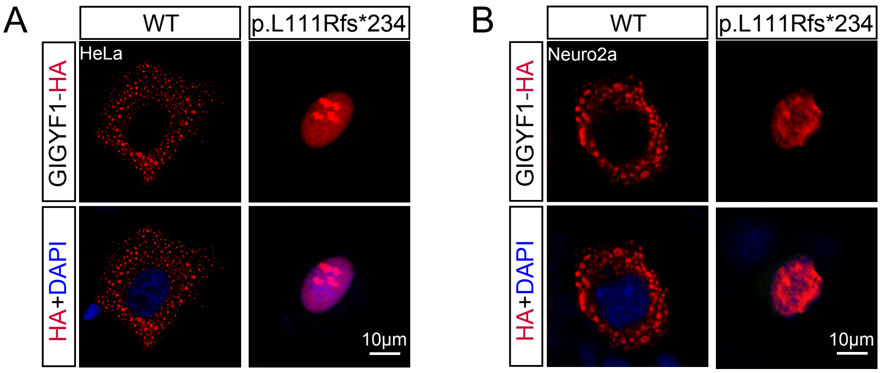

Supplemental Figure 3. The recurrent LGD site p.L111Rfs*234 shows abnormal localization in HeLa (**A**) and Neuro2a (**B**) cells. The wild-type (WT) GIGYF1 is mainly located in the cytoplasm. However, the mutant is mainly located in the nuclei. Scale bars represent 10 μm.

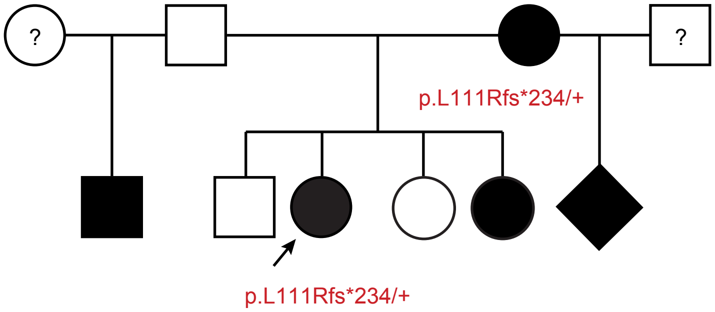

Supplemental Figure 4. Pedigree plot of a family with *GIGYF1* p.L111Rfs*234 variant and substantial family history of ASD/NDD recruited through GeneMatcher.

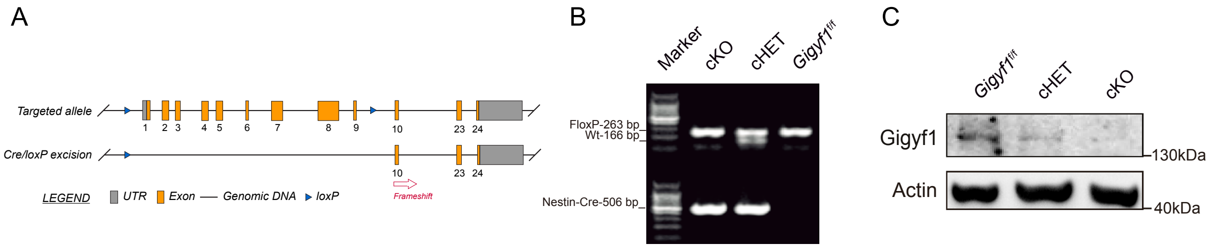

Supplemental Figure 5. Construction and identification of Gigyf1 conditional knockout mice. (A) Schematic overview of Gigyf1 knockout strategy. *Gigyf1* gene exons 1-9 were bounded by loxP sites. Gigyf1$^{f/w}$-CreNestin (cHET) and Gigyf1$^{f/f}$-CreNestin (cKO) mice were generated by crossing floxed Gigyf1 mice (*Gigyf1*$^{f/f}$, Control) with Nestin-Cre mice. **(B)** Genotype identification of *Gigyf1*$^{f/f}$ (Control), *Gigyf1*$^{f/w}$-Cre^{Nestin} (cHET) and *Gigyf1*$^{f/f}$-Cre^{Nestin} (cKO) mice. **(C)** Detection of endogenous Gigyf1 expression in the cortex of *Gigyf1*$^{f/f}$, *Gigyf1*$^{f/w}$-Cre^{Nestin} (cHET) and *Gigyf1*$^{f/f}$-Cre^{Nestin} (cKO) mice.

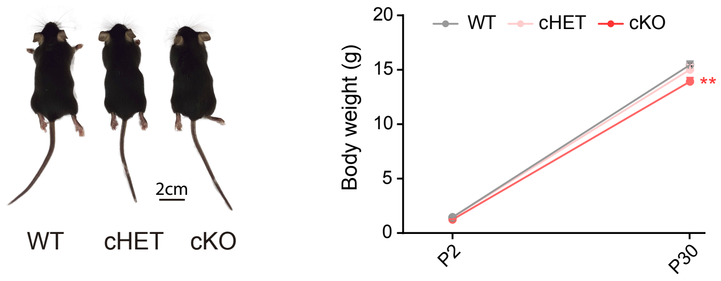

Supplemental Figure 6. Dorsal image and body weight comparisons of G*igyf1*$^{f/f}$, cHET, and cKO mice at P30. The right plot shows the comparisons of body weight between G*igyf1*$^{f/f}$ (n=10), cHET (n=10), and cKO (n=6) mice at P2 and P30. Statistic data are analyzed using two-way ANOVA. Data values in all statistic graphs represent mean ± SEM. **, p<0.01.

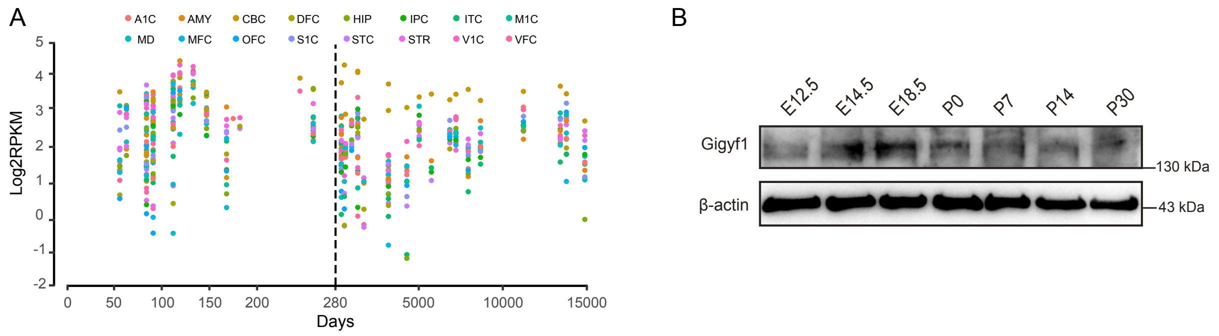

Supplemental Figure 7. Dynamic expression mode of GIGYF1 in Human and mouse development brains. (A) Expression of GIGYF1 during brain development periods in different brain regions. The x axis is the age of samples in days and y axis is the log2-transformed RPKM of GIGYF1. A1C, primary auditory cortex; AMY, amygdaloid complex; CBC, cerebellar cortex; DFC, dorsolateral prefrontal cortex; HIP, hippocampus; IPC, inferior parietal cortex; ITC, inferolateral temporal cortex; M1C, primary motor cortex; MD, mediodorsal nucleus of thalamus; MFC, medial prefrontal cortex; OFC, orbital frontal cortex; S1C, primary somatosensory cortex; STC, superior temporal cortex; STR, striatum; V1C, primary visual cortex; VFC, ventrolateral prefrontal cortex. The dashed line indicates the birthday. **(B)** Profiling of Gigyf1 protein expression in the cerebral cortex during the embryonic and postnatal period. Gigyf1 was highly expressed in early embryonic development period and decreased in postnatal period.

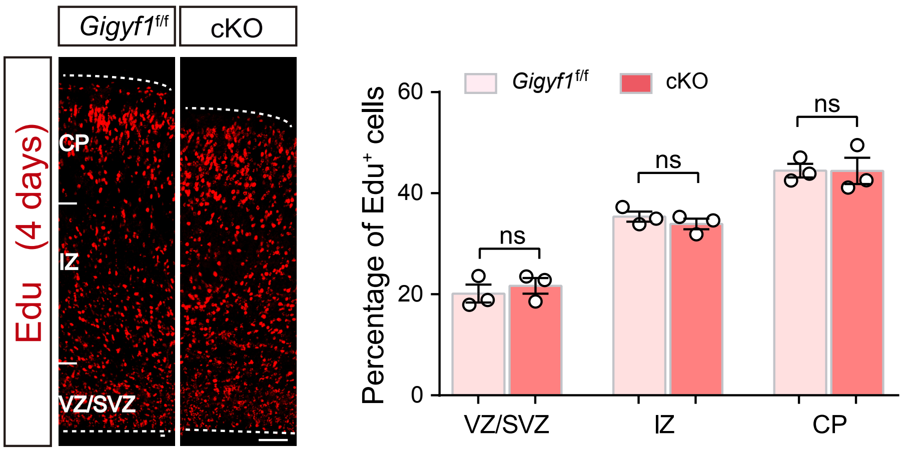

Supplemental Figure 8. Migration analysis of the distribution of EdU$^+$ neurons at E18.5. Representative IH images of EdU single-stained cortical sections of G*igyf1*$^{f/f}$ and cKO mice at E18.5 are shown. Distribution of EdU$^+$ cell in different cortical zones 4 days after injecting EdU at E14.5 were calculated and compared. Scale bar represents 50 μm. Statistic data are analyzed by two-way ANOVA. Data values in all statistic graphs represent mean ± SEM. ns, not significant.

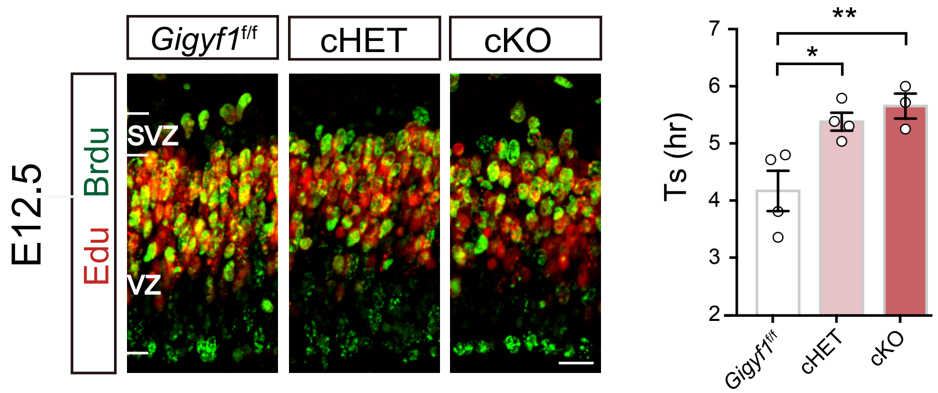

Supplemental Figure 9. S phase sequential labeling analysis of NPC at E12.5. Representative IH images of EdU/Brdu double-stained cortical sections of G*igyf1*$^{f/f}$, cHET, and cKO mice at E12.5 are shown. EdU$^+$Brdu$^+$ cells are cells in S phase (Scells). S phase durations (Ts, hours) of G*igyf1*$^{f/f}$, cHET, and cKO mice were calculated (Ts=Ti/(Lcells/Scells)) and compared. Scale bar represents 50 μm. Statistic data are analyzed using one-way ANOVA. Data values in all statistic graphs represent mean ± SEM. *, $p < 0.05$; **, $p < 0.01$.

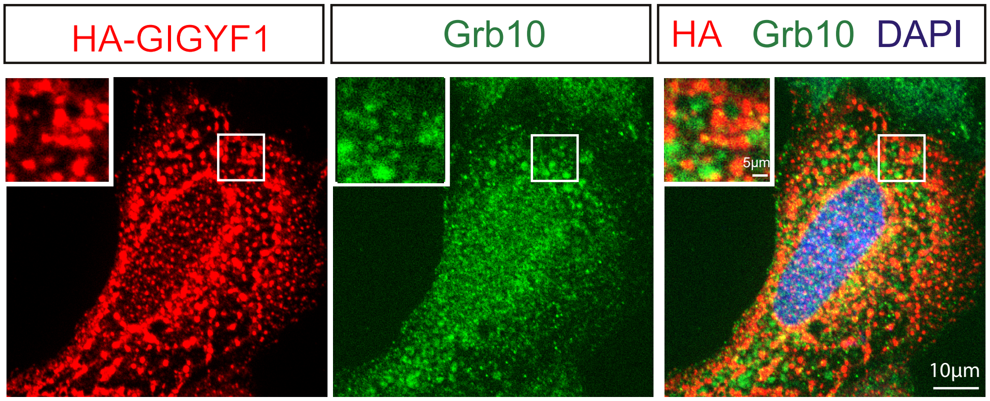

Supplemental Figure 10. Double immunofluorescence of GIGYF1 and GRB10 in HeLa cells. GIGYF1 is co-localized with GRB10 in cells expressing HA-tagged GIGYF1 and immunostained with antibodies to GRB10. Scale bar represents 10 μm. The area of the white box is enlarged in the upper left corner of the picture. Scale bars of insets represent 5 μm.

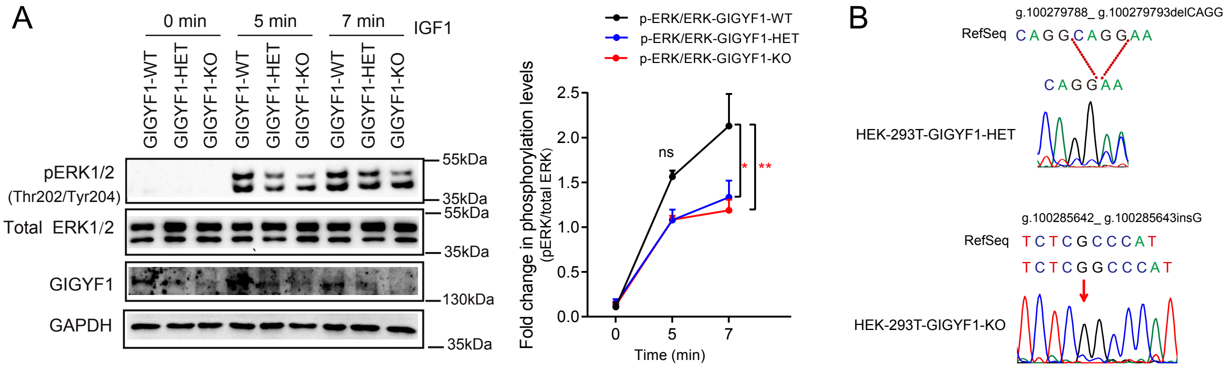

Supplemental Figure 11. Haploinsufficiency or knockout of GIGYF1 in HEK293T cells decreases the phosphorylation of ERK. (A) Immunoblots of the whole cell lysate showing phosphorylation of ERK1/2 (pERK1/2) and ERK1/2 at different duration of IGF-1 stimulation in GIGYF1 WT, heterozygous (HET) and KO cells. Statistic data are presented as fold changes in phosphorylation levels compared to total levels quantified by densitometry and analyzed using two-way ANOVA. Data values in all statistic graphs represent mean ± SEM. *p<0.05, **p<0.01, ns represents significant. **(B)** LentiCRISPR V2-mediated GIGYF1 HET and KO cells were detected by Sanger sequencing and compared with the reference sequence.

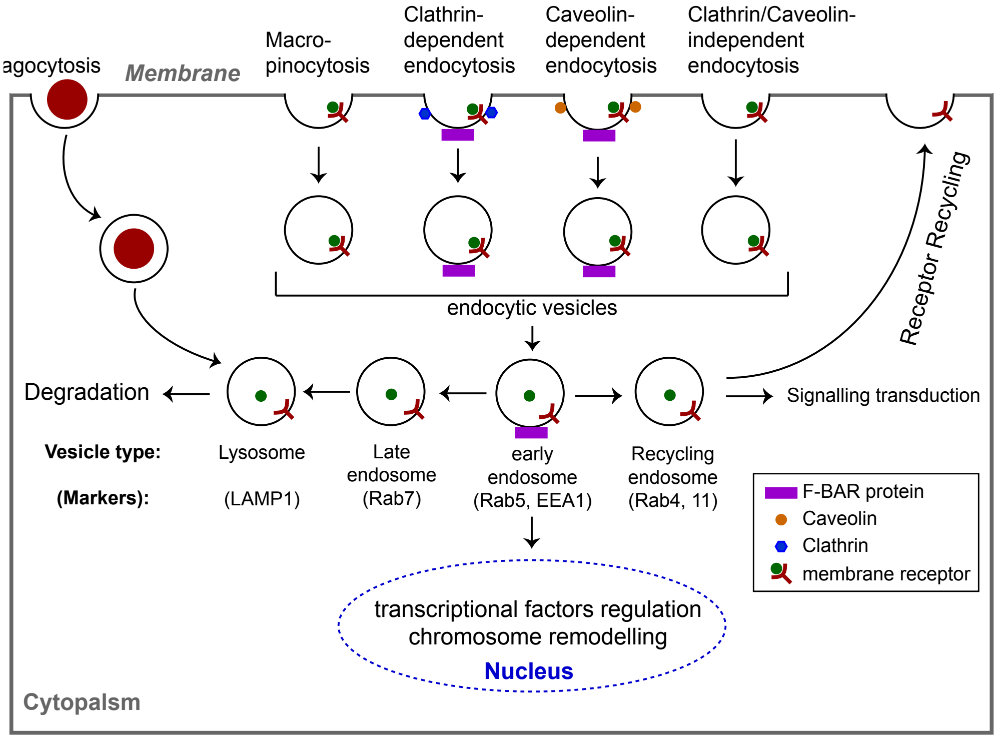

Supplemental Figure 12. Schematic diagram of endocytosis. Ligands bind to receptors in cell membranes to induce endocytosis by receptor-dependent or receptor-independent mechanisms, and then forming phagosomes or endocytic vesicles wrapped by Clathrin or caveolin. Once pinocytosis, internalized vesicles are transported to the early endosomes (Rab5 and EEA1). Early endosomes can be transported to late endosomes (Rab7), followed by lysosomes for degradation. Early endosomes can also be transported to recycling endosomes (Rab4 and Rab11) for recycling to plasma membrane. Meanwhile, early endosomes can be used for transcription factor regulation or chromatin remodeling in the nucleus.

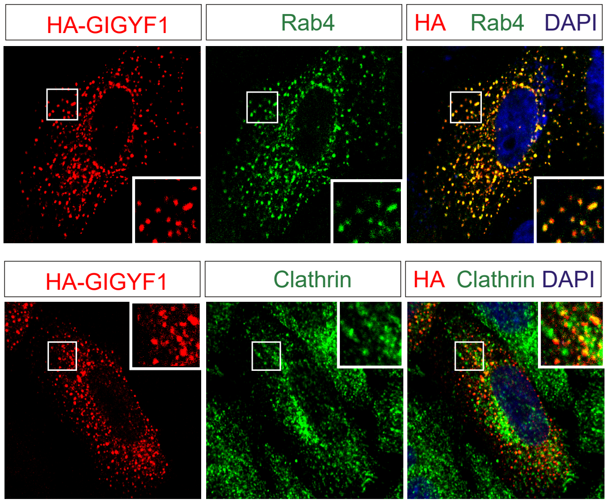

Supplemental Figure 13. Co-localization of GIGYF1 and endosome markers in HeLa cells. GIGYF1 is strongly co-localized with Rab4 and partially co-localized with Clathrin, EEA1, and Rab5A in cells expressing HA-tagged GIGYF1 and immunostained with antibodies Clathrin, EEA1, Rab5A and Rab4. Scale bars represent 10 μm.The area of the white box is enlarged in the upper right corner of the picture. Scale bars of insets represent 5 μm.

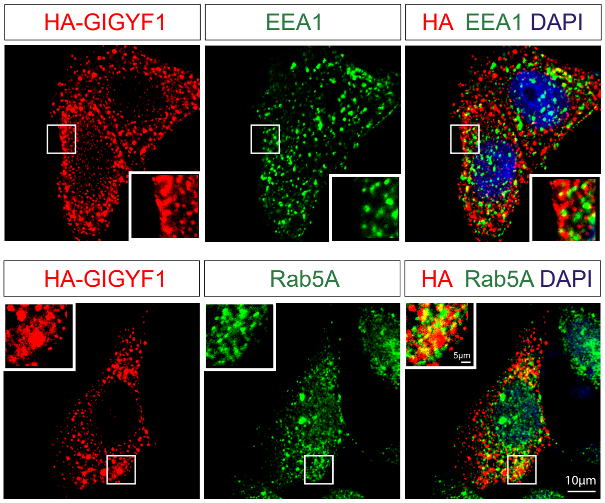

Supplemental Figure 14. Co-localization of GIGYF1 and endosome markers in HeLa cells. GIGYF1 is not co-localized with caveolae, Rab11, Rab7, and Lamp1 in cells expressing HA-tagged GIGYF1 and immunostained with antibodies to caveolae, Rab11, Rab7, and Lamp1. Scale bars represent 10 μm.

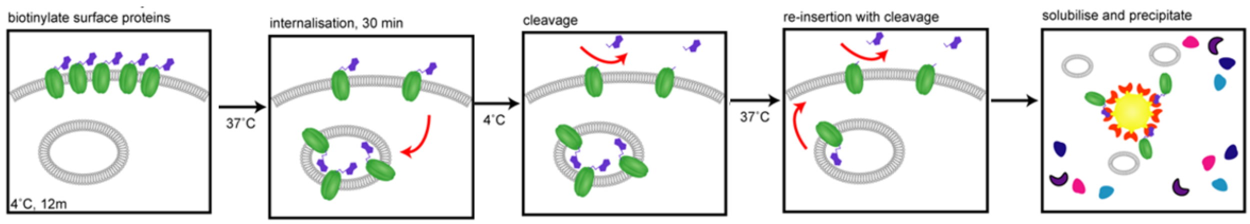

Supplemental Figure 15. Workflow of Surface biotinylation recycling assay.

Surface labelled with Sulfo-NHS-S-S-biotin. The labelling reaction was quenched, and then cells were incubated to endocytosis. Remaining surface biotin was cleaved. Cells were incubated for a second time to recycle and surface stripped for a second time. Cells were lysed and incubated with NeutrAvidin Agarose. Proteins were immunoblotted by western blot. The diagram was derived from https://doi.org/10.6084/m9.figshare.816936.v1.

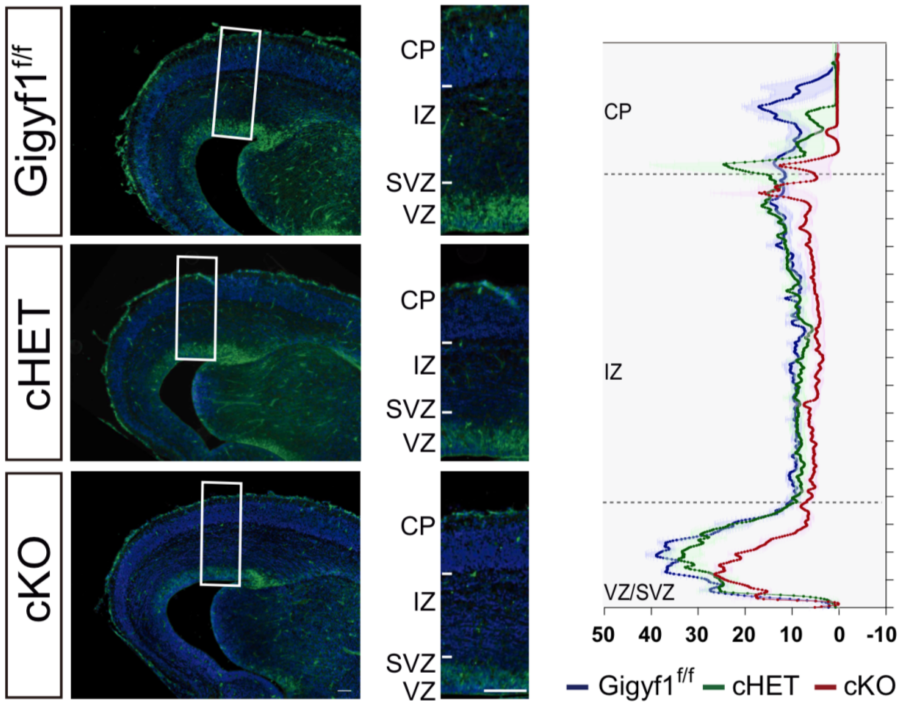

Supplemental Figure 16. pERK level in *Gigyf1* knockout mice cortical zones. Representative IH images of pERK1/2 in the neocortex of G*igyf1*$^{f/f}$, cHET, and cKO mice at E14.5. Immunofluorescence intensity of pERK1/2 was calculated and compared. The area of the white box is enlarged on the right. Scale bar represents 100 µm.

Supplementary Methods for Behavioral tests

Three-chamber test. A three-chamber test was conducted to analyze whether GIGYF1 knockout mice had abnormal social and socially oriented behavior by comparing the time spent exploring objects with that of unfamiliar mice. Preparation: 2 black bottle caps of similar size to the test mice; 2 wild-type C57BL6 mice from non-identical litters, of the same age, sex and weight as possible and from the same litter if possible. The mice were allowed to acclimatize in an intermediate chamber for 5 minutes prior to the start of the test. In the first round, black caps were placed on both the left and right sides of the cage; in the second round, the cap on one side was replaced with a B6 wild mouse; in the third round, the cap on the other side was replaced with another B6 wild mouse and the previous round was left untouched. Each round is tested for 10 minutes, and the box is wiped with 25% alcohol between each round to remove the odor. A computerized video software system automatically records the route and time taken by the mice between the three chambers, recording the number of times and the time taken by the mice to explore the left and right cages in each round.

Marbles bury test. All cages were made from standard mouse cages, and a bedding of approximately 5cm thickness was placed in the cages. The glass marbles were soaked in 25% ethanol, washed thoroughly with deionized water, and placed on the surface of the bedding, with the marbles arranged neatly in rows of 5 in 4 columns. The recording apparatus was set up and the experimental mice were placed in the arranged experimental set-up with the lid closed. The number of glass marbles buried 2/3 of the way through each experiment was recorded for 30 minutes, and the mean number of marbles buried was calculated for each genotype of mice.

Digging, rearing and grooming test. The experimental set-up is a glass cylinder made of transparent glass. The experimental apparatus is 30 cm high and 20 cm in diameter. The apparatus is placed on a cleared floor and a bedding material of about 1 cm thickness is placed on the bottom. A video camera is set up and the mice are placed in the set-up. After 5 minutes of acclimatization, the camera is started and the number and duration of spontaneous hair stroking behavior of the mice are recorded by video for 10 minutes.

Morris water-maze test. The experiment consisted of two main parts: the Hidden Platform Test and the Probe Trains. The learning test was conducted for 4 days, with the mice being placed in the water once a day from each of the 4 entry points facing the wall of the pool, and the time taken to find the hidden platform was recorded. The spatial exploration test is performed by removing the platform after the learning test and placing the mouse in the pool from any one of the entry points. Irrespective of the trial performance, mice were guided to the platform and allowed to remain there for at least 15s. Spatial exploration test: After completing the learning trial, the platform is removed. The time spent in the target quadrant, the distance in the target quadrant, the number of times the mouse crossed the target quadrant, the swimming speed were recorded, and this data was used to determine the spatial orientation of the mouse and the pattern of change during spatial exploration. The number of times the mouse crossed the original location of the platform was recorded by marking the original location of the platform with a circular ring and displaying it on the computer screen(1).

Light-dark box test. The light-dark box test set-up is a black and white box custom made in the laboratory according to experimental standards in plexiglass. The set-up is 44 cm long, 21 cm wide and 21 cm high. The light box is 28 cm long, colorless, and transparent, without a lid; the dark box is 14 cm, black with a lid and has a volume ratio of 2:1. A small door 10 cm wide and 5 cm high

is provided between the light and dark boxes. The software and the camera were turned on and it was tested that the experimental set-up is the same as the area of the bright and dark boxes predetermined in the software and closed the small door with plexiglass. At the beginning of the experiment, the experimental mice were placed in the dark box experimental set-up and the number of times the mice were shuttled between the light and dark boxes and the duration of their stay in the light and dark boxes within 10 minutes were recorded. The distance and time of staying in the dark and light boxes were used to reflect their anxiety.

Elevated plus maze test. The elevated cross-maze experimental set-up is based on the design of the experimental set-up by Autry et al. The device is made of plexiglass and has two open arms and two closed arms with four arms in a crossed position, the open arm is 30 cm long, 5 cm wide and 0.5 cm high, and the closed arm is 30 cm long, 5 cm wide and 15 cm high. The height of the device was set at 50 cm. The software and the camera were turned on and it was tested that the experimental set-up has the same area as the predetermined open and closed arms in the software. At the beginning of the experiment, the experimental mice were placed in the middle area of the cross and the number of shuttles and dwell time of the mice in the open and closed arms within 10 minutes were recorded. The distance the mice moved in the open and closed arms and the length of time they stayed in the different areas were used to reflect their anxiety.

Open field test. The open-field test set-up is a square box custom made in the laboratory from plexiglass according to experimental standards, the set-up is 72 cm long, 72 cm wide and 40 cm high. The bottom of the box is made up of 16 identical small squares, with the inner 4 squares in the middle set as the middle area and the rest set as the outer perimeter area. The software and camera are switched on and the experimental set-up is tested to see if it matches the predetermined middle and peripheral areas in the software. The mice were placed in the middle of the

experimental apparatus in the open field and the time and number of movements in the middle and peripheral areas were recorded over a 10-minute period. The time spent in the middle area and the number of shuttles were used to reflect their anxiety.

Novel-object recognition test. Two objects in an environment with three objects A, B and C where objects A and B are identical and object C is completely different from objects A and B. The procedure is as follows: 1. Start the training by placing the two objects A and B on the left and right ends of a sidewall with the mice's backs facing the two objects into the field, and the mice's noses should be the same length from the two objects. The mice are placed for 10min. Immediately after placement, the video equipment is switched on and the experimenter immediately leaves the test room to record the mice's contact with the two objects, including the number of times the nose or mouth touches the objects and the time spent exploring within 2-3cm of the objects. 2. 24 hours later, put the mice back into the original mouse box where they were kept, replace object B in the field with object C. Still place the mice with their backs to the two objects, with the tips of their noses at the same distance according to the two objects, and observe for 10min, again using the video recording equipment to record the mice's contact with the objects (2).

References

1. Vorhees CV, and Williams MT. Morris water maze: procedures for assessing spatial and related forms of learning and memory. *Nat Protoc.* 2006;1(2):848-58.
2. Lueptow LM. Novel Object Recognition Test for the Investigation of Learning and Memory in Mice. *J Vis Exp.* 2017(126).

Full Uncut Blots

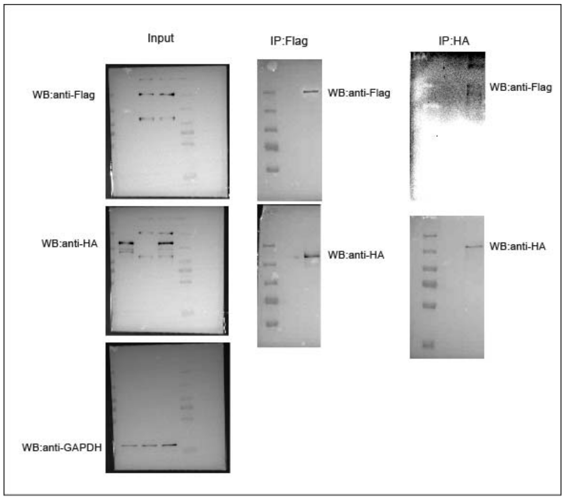

Full unedited Blots for Figure 5A

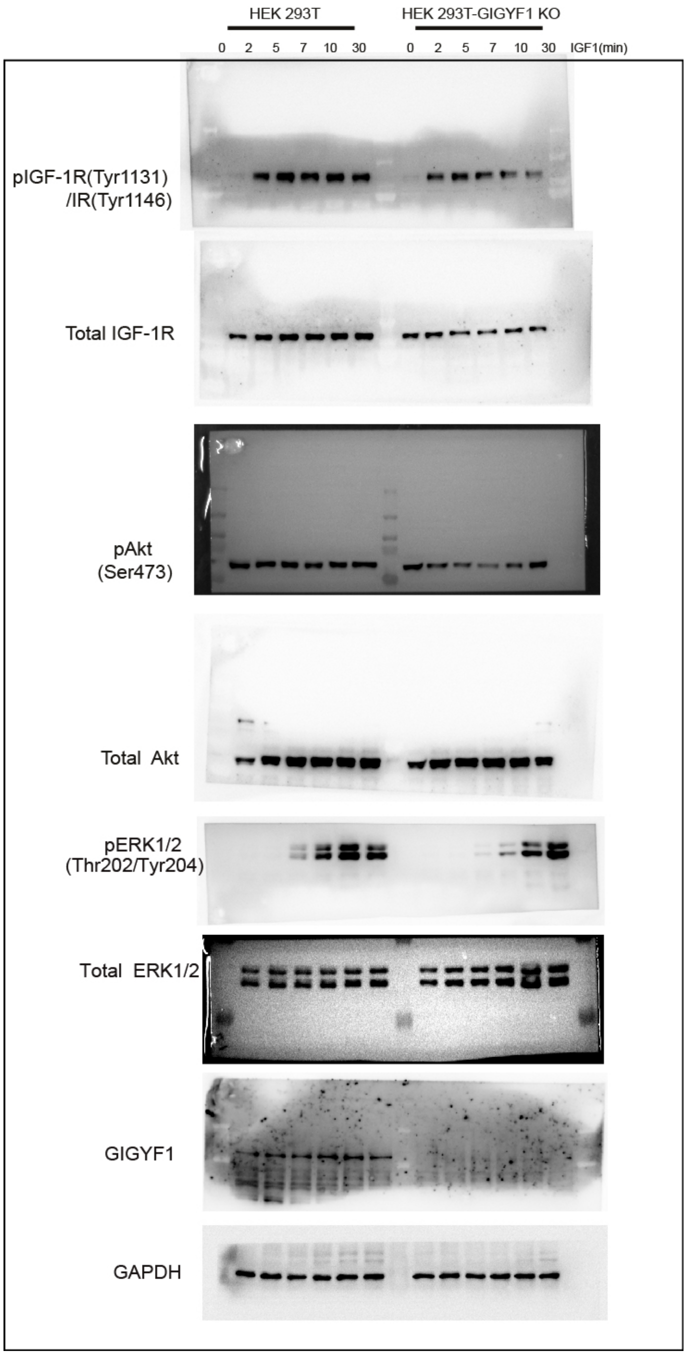

Full unedited Blots for Figure 5C

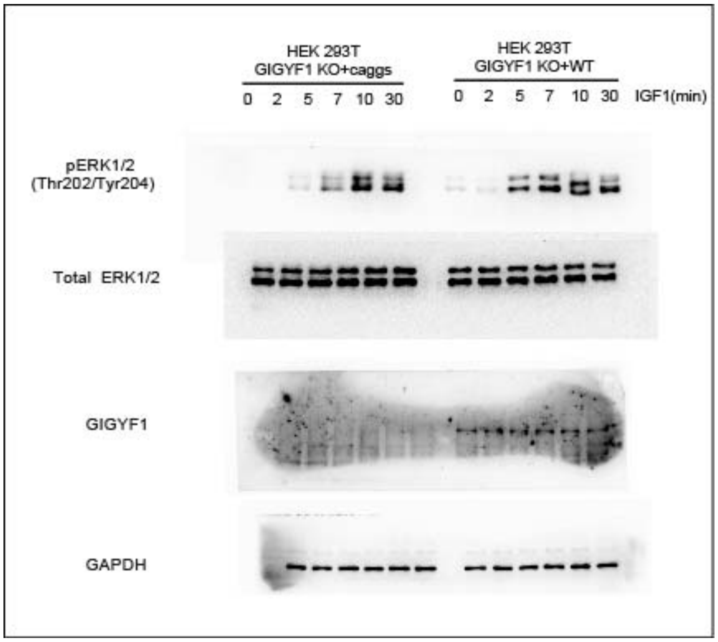

Full unedited Blots for Figure 5D

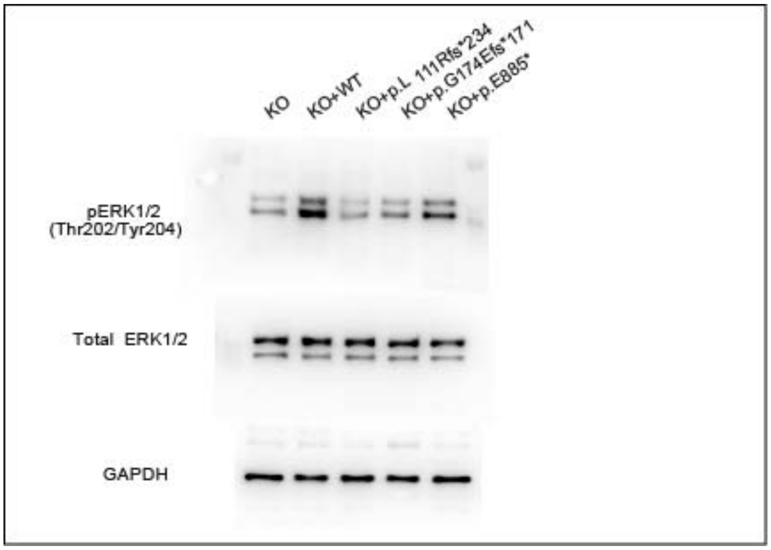

Full unedited Blots for Figure 5E

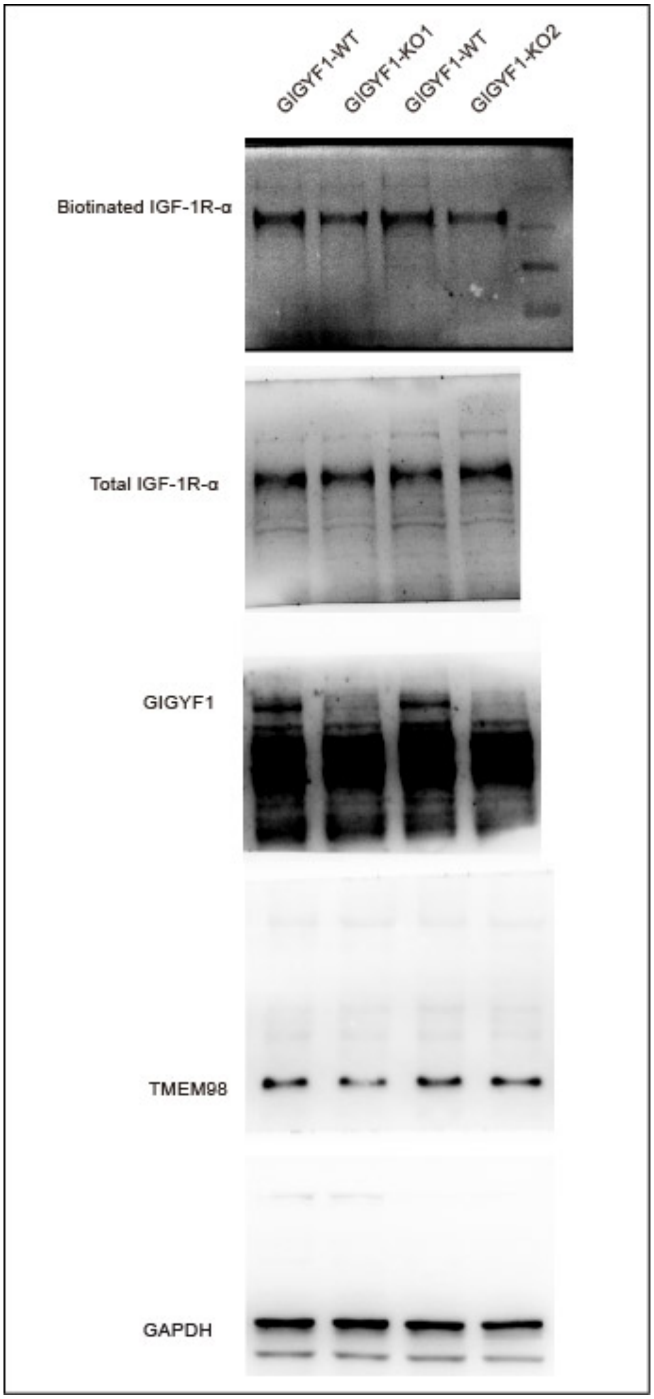

Full unedited Blots for Figure 5F

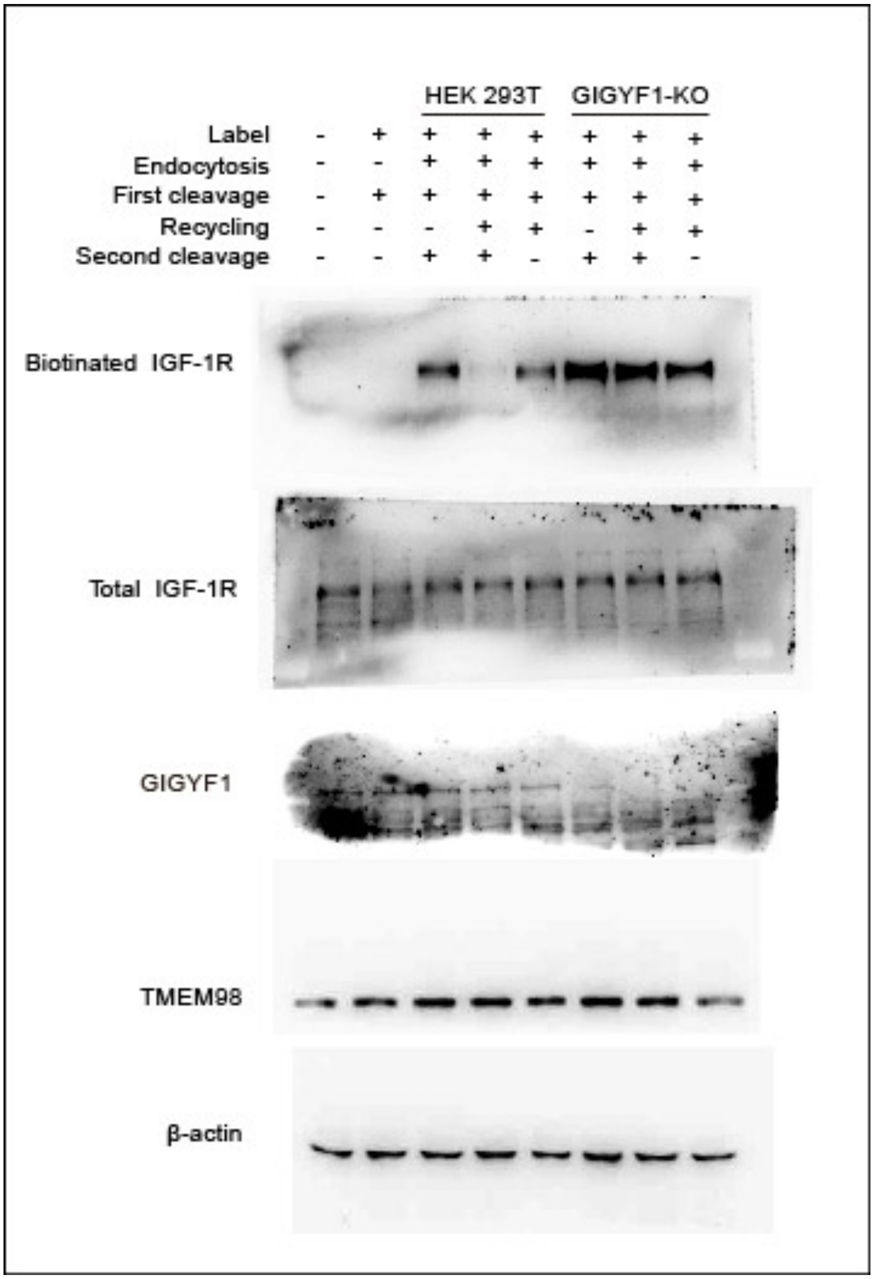

Full unedited Blots for Figure 5G

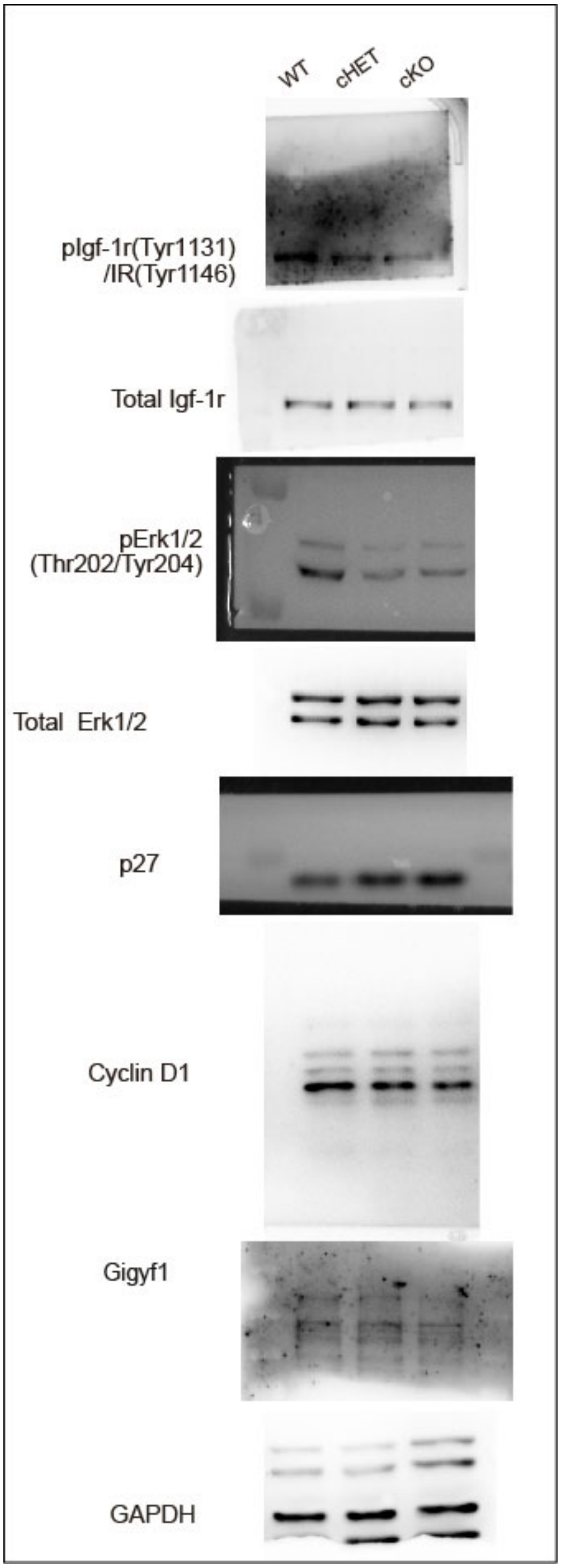

Full unedited Blots for Figure 6A

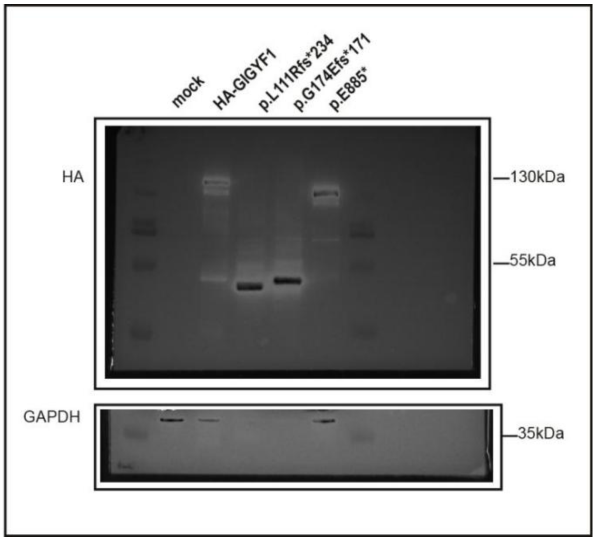

Full unedited Blots for Supplemental Figure 2

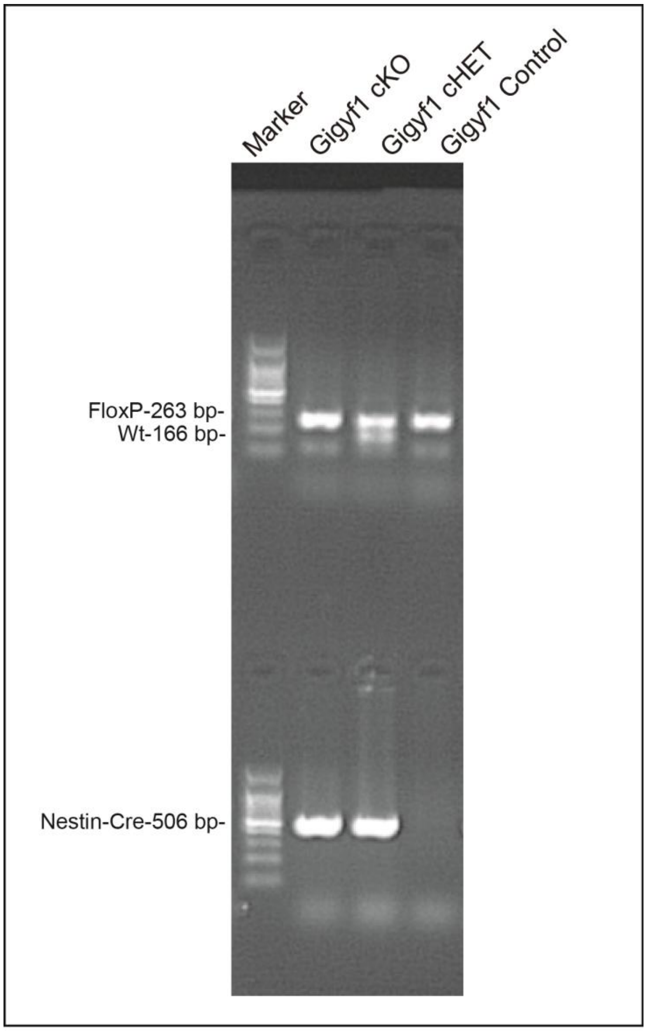

Full unedited Blots for Supplemental Figure 5B

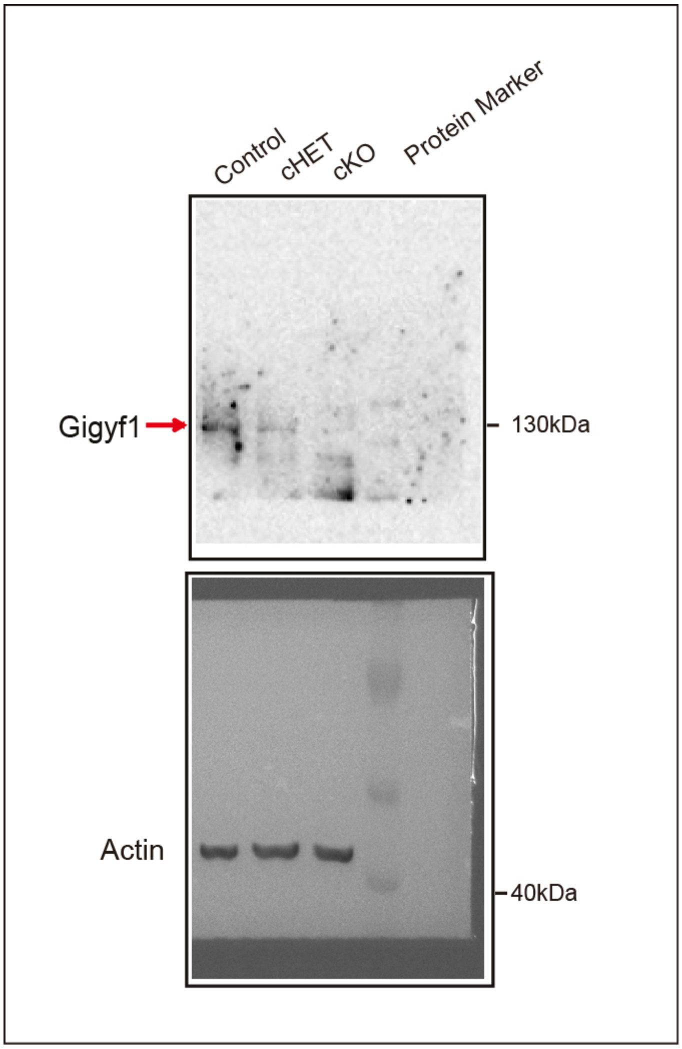

Full unedited Blots for Supplemental Figure 5C

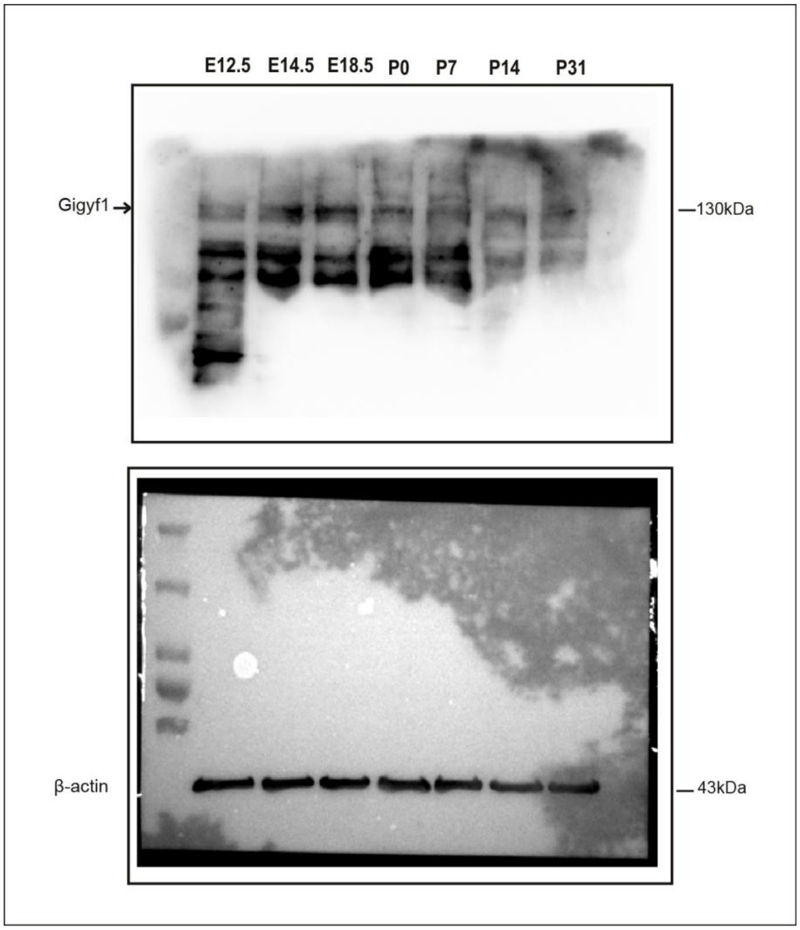

Full unedited Blots for Supplemental Figure 7B

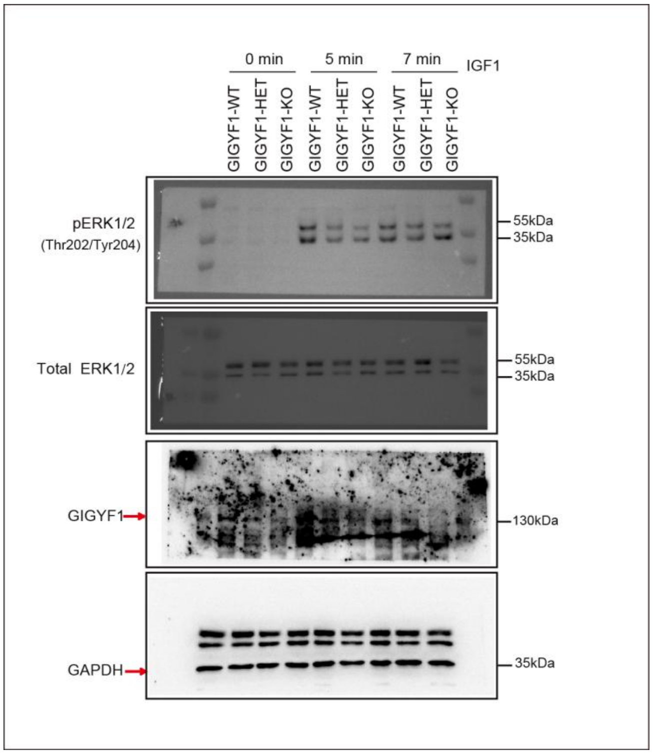

Full unedited Blots for Supplemental Figure 11A
